# 4-(1-Methylamino)ethylidene-1,5-disubstituted pyrrolidine-2,3-diones: synthesis, anti-inflammatory effect and in silico approaches

**DOI:** 10.3762/bjoc.21.65

**Published:** 2025-04-24

**Authors:** Nguyen Tran Nguyen, Vo Viet Dai, Luc Van Meervelt, Do Thi Thao, Nguyen Minh Thong

**Affiliations:** 1 The University of Danang-University of Science and Education, Danang 550000, Vietnam; 2 Biomolecular Architecture, Department of Chemistry, KU Leuven, Celestijnenlaan 200F, B-3001 Leuven, Belgium; 3 Institute of Biotechnology, Vietnam Academy of Science and Technology (VAST), Hanoi 10072, Vietnam

**Keywords:** anti-inflammatory pyrrolidine-2,3-dione derivatives, iNOS, pyrrolidine-2,3-dione derivatives, pyrrolidine-2,3-diones, pyrrolidine-2,3-diones targeting reversible transimination reaction

## Abstract

Pyrrolidine-2,3-diones are important intermediates in the synthesis of numerous nitrogen-containing heterocycles which possess a broad spectrum of biological and pharmacological activities. In this article, we report the synthesis of 4-(1-methylamino)ethylidene-1,5-disubstituted pyrrolidine-2,3-diones via a reversible transimination reaction between Schiff’ base (C=N) linkage-containing pyrrolidine-2,3-dione derivatives and methylamine with yields of 80 to 92%. In addition to nuclear magnetic resonance spectroscopy, the structure of 4-(1-methylamino)ethylidene-1,5-diphenylpyrrolidine-2,3-dione (**5a**) was also verified through single-crystal X-ray diffraction. Furthermore, the synthesized molecules were evaluated for compliance with established drug-likeness rules (Lipinski, Veber, Ghose, Egan, and Muegge), as well as ADMET properties. All compounds satisfied these criteria, indicating favorable oral bioavailability. Molecular docking analysis showed that compounds **5a**–**e** act as ligands for inducible nitric oxide synthase (iNOS), especially with Cys200 and Ser242 via hydrogen bonds. In addition, van der Waals interactions also contribute to the stabilization of the ligand–iNOS complexes. In particular, 4-(1-methylamino)ethylidene-5-phenyl-1-(3-nitrophenyl)pyrrolidine-2,3-dione (**5e**) exhibited the strongest binding affinity (−9.51 kcal/mol) and demonstrated significant inhibitory activity against nitric oxide (NO) production, with an IC_50_ value of 43.69 ± 5.26 µM. The presence of an electron-withdrawing group (-NO_2_ group) on the benzene ring at the 1-position of the pyrrolidine-2,3-dione subunit in compound **5e** may be responsible for the observed high inhibition activity due to the enhancement and optimization of hydrogen bonding with Cys200. These results underscore the potential of 4-(1-methylamino)ethylidenepyrrolidine-2,3-diones, especially compound **5e**, as promising scaffolds for the development of anti-inflammatory agents targeting iNOS-related pathologies.

## Introduction

Nitric oxide (NO) is an important signaling molecule in numerous physiological processes such as neuronal tranmission, immue response, inflammatory response, respiratory, vasodilation, apoptosis, tumor growth, and cardiovascular system [1–2]. Nitric oxide (NO) is released as product of the NADPH and oxygen-dependent oxidation of ʟ-arginine to ʟ-citrulline under the catalysis of the enzyme nitric oxide synthase (NOS) [3]. There are three distinct isoforms of NOS named according to their site of existence and include inducible NOS (iNOS), endothelial NOS (eNOS), and neuronal NOS (nNOS) [4]. For example, upon bacterial infection in humans, immune cells express iNOS which promotes the release of NO causing inflammation [5] and NO supports defense to eliminate invading bacteria via the inhibition of metabolic enzymes and DNA destruction. However, overexpression of iNOS results in the overproduction of NO which is associated with septic shock and tissue damage [6]. Therefore, inhibition of iNOS could be considered a potential therapeutic strategy for controlling inflammatory diseases.

**Figure 1:**
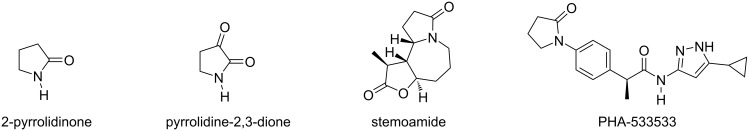
Natural products and synthetic medicinal compounds containing a 2-pyrrolidinone subunit.

There have 2-pyrrolidinone subunit-containing compounds which exhibited promising biological activities [7]. For example, stemoamide extracted from *Stemona tuberosa* Lour, a Chinese traditional medicine, has been used in the treatment of asthma and tuberculosis [8–9]. (2*S*)-*N*-(5-Cyclopropyl-1*H*-pyrazol-3-yl)-2-[4-(2-oxo-1-pyrrolidinyl)phenyl]propanamide (PHA-533533), a synthetic compound containing a 2-pyrrolidinone ring, has been investigated in cancer treatment (Figure 1) [10]. In addition, molecular docking simulation and in vitro studies have shown that some pyrrolidine-2,3-dione derivatives could inhibit biomacromolecules (DNA, BSA, mPGES-1 or CDKs) and consequently, enable them to be candidates for Alzheimer's disease [11], anti-inflammatory [12–17], and antitumor drug discovery [12]. Furthermore, pyrrolidine-2,3-diones are also important intermediates in the synthesis of 2-pyrrolidinone ring containing bioactive medicinal compounds [18].

In this article, we report the accidental synthesis of 4-(1-methylamino)ethylidene-1,5-disubstituted pyrrolidine-2,3-dione derivatives via the transimination reaction between 4-[1-(4-methoxybenzyl)amino]ethylidene-1,5-disubstituted pyrrolidine-2,3-diones and methylamine. All studied compounds were successively investigated to what extent they inhibit nitric oxide (NO) production in LPS-stimulated RAW264.7 macrophages. In addition, a molecular docking simulation was performed to examine the binding interactions of these derivatives with the enzyme inducible nitric oxide synthase (iNOS), compared to dexamethasone used as a reference. Furthermore, ADMET (absorption, distribution, metabolism, excretion, and toxicity) predictions were performed to assess their drug-likeness and pharmacokinetic properties, while density functional theory (DFT) calculations provided insights into their electronic properties, including reactivity and stability. This comprehensive approach, integrating synthesis, biological evaluation, and computational methods, highlights the potential of 4-(1-methylamino)ethylidene-1,5-disubstituted pyrrolidine-2,3-diones as scaffolds for the development of anti-inflammatory agents targeting iNOS-related pathologies.

## Results and Discussion

### Synthesis of 4-(1-methylamino)ethylidene-1,5-disubstituted pyrrolidine-2,3-diones **5a–e**

**Scheme 1:**
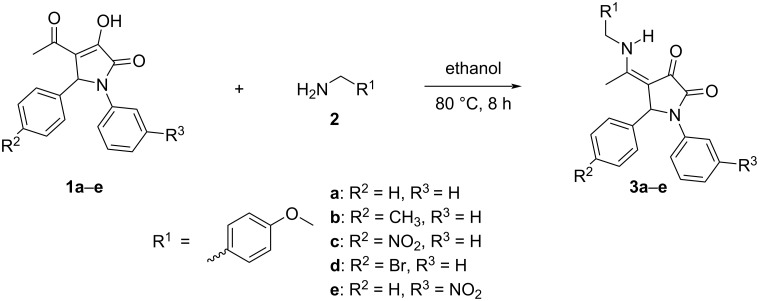
Synthesis of 4-[1-(4-methoxybenzyl)amino]ethylidene-1,5-disubstituted pyrrolidine-2,3-diones **3a–e**.

The reaction between 4-acetyl-3-hydroxy-1,5-disubstituted-3-pyrroline-2-ones **1a–e** and 4-methoxybenzylamine (**2**) in absolute ethanol yielded 4-[1-(4-methoxybenzyl)amino]ethylidene-1,5-disubstituted pyrrolidine-2,3-diones **3a–e** (Scheme 1) [19–21]. In addition to nuclear magnetic resonance spectroscopy (1D, 2D NMR), the structure of **3a** has also been proven via single-crystal X-ray diffraction [19].

**Scheme 2:**
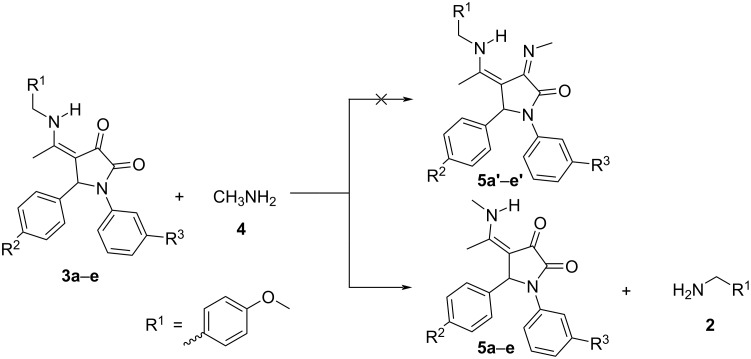
Synthesis of 4-(1-methylamino)ethylidene-1,5-disubstituted pyrrolidine-2,3-diones **5a–e**.

According to a previous publication, the condensation between pyrrolidine-2,3-diones and an amine as nucleophile normally occurred at the 3-position of nitrogen-containing heterocyclic ring which results in the corresponding enamine product [18]. However, the reaction between pyrrolidine-2,3-dione derivatives **3a–e** and methylamine (**4**) resulted in the formation of compounds **5a–e** instead of the expected 3-substituted products **5a’–e’** (Scheme 2). It is undoubtedly the case that the reaction between 1,4,5-trisubstituted pyrrolidine-2,3-diones **3a–e** and methylamine (**4**) has occurred at the exocyclic sp^2^-hybridized carbon atom, leading to the substitution of the 4-methoxybenzylamino group by a methylamino group. Therefore, the presence of substituents (electron-donating or electron-withdrawing groups) on the benzene rings attached to the 1- and 5-positions of the pyrrolidine-2,3-diones **3b–e** are not expected to affect the scope of the reaction (Scheme 2, Table 1). When the reaction between **3a** (1 equiv) and methylamine (**4**) (4 equiv, 40% in water) was carried out in absolute ethanol (0.3 mL) at reflux, 4-(1-methylamino)ethylidene-1,5-diphenylpyrrolidine-2,3-dione (**5a**) was obtained in 80.8% yield. Gratifyingly, substrate **3a** is well soluble in methylamine solution (40% in water) and conducting the reaction between **3a** (1 equiv) and methylamine (**4**) (40% in water, 0.3 mL, 47 equiv), without any other solvent added, the yield of product **5a** increased to 92.2% (Table 1). Therefore, excess methylamine (40% in water, 47 equiv), served as both the solvent and nucleophilic reactant in the above reaction.

**Table 1:** Synthesis of 4-(1-methylamino)ethylidene-1,5-disubstituted pyrrolidine-2,3-diones **5a–e**.

Entry	R^2^	R^3^	Solvent	Ratio **3a–e**:**4** (equiv)	Product	Yield (%)

1	H	H	ethanol	1:4	**5a**	80.8
2	H	H	methylamine 40% in water	1:47	**5a**	92.2
3	CH_3_	H	methylamine 40% in water	1:47	**5b**	89.4
4	NO_2_	H	methylamine 40% in water	1:47	**5c**	90.1
5	Br	H	methylamine 40% in water	1:47	**5d**	85.3
6	H	NO_2_	methylamine 40% in water	1:47	**5e**	88.3

In the ^1^H NMR spectrum of **3a**, there are two doublets of doublets at 4.40 ppm and 4.49 ppm corresponding to two methylene protons (CH_2_) which are diastereotopic and therefore, show geminal coupling to each other [19]. However, in the ^1^H NMR spectrum of product **5a** the above peaks were absent and the appearance of a new doublet at 2.76 ppm representing three protons from the methyl group (CH_3_) is observed. In addition, the 2D ^1^H–^1^H COSY NMR spectrum of **5a** exhibited spin–spin coupling between the secondary amino proton and the proton resonance at 2.76 ppm (see Supporting Information File 1). Therefore, the doublet resonance signal at 2.76 ppm correlates to three protons from the methyl group (CH_3_) directly bonded to the nitrogen atom of the secondary amino group (NH). Furthermore, the 2D ^1^H–^13^C HMBC NMR spectrum of **5a** also exhibited the correlation between protons of two methyl groups and the same carbon atom resonance at 165.52 ppm, respectively (see Supporting Information File 1). Consequently, the signal at 165.52 ppm in the ^13^C NMR spectrum corresponds to the sp^2^-hybridized carbon atom directly bonded to the nitrogen atom of the secondary amino group of compound **5a**.

**Scheme 3:**
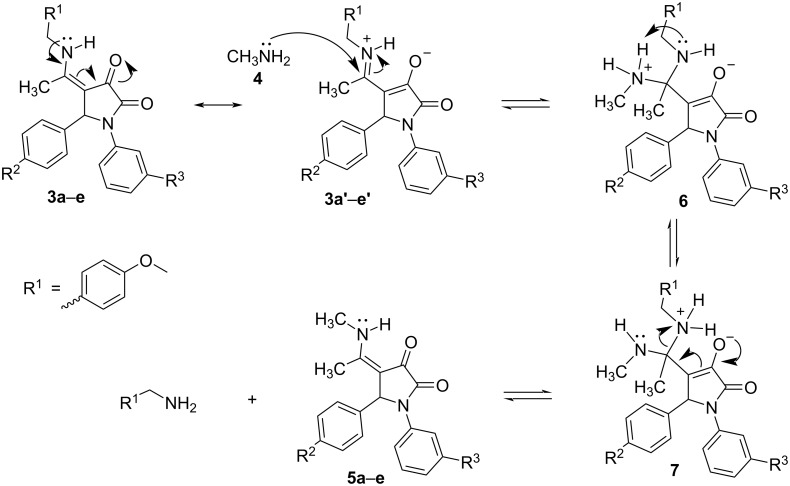
Proposed mechanism for the reaction between 4-[1-(4-methoxybenzyl)amino]ethylidene-1,5-disubstituted pyrrolidine-2,3-diones **3a–e** and methylamine (**4**).

In the structure of each pyrrolidine-2,3-dione derivative **3a–e**, there is an α,β-unsaturated ketone moiety in which the π systems of the C=C and C=O bonds could overlap each other to yield an extended conjugated system. Furthermore, the delocalization of a lone pair of electrons on the secondary nitrogen atom will bring about a new π bond with its adjacent carbon atom in the resonance form **3a’–e’**. Consequently, pyrrolidine-2,3-diones **3a–e** and their resonance forms are stabilized by electron delocalization and charge separation. In the resonance forms **3a’**–**e’**, the 4-methoxybenzylamino group is covalently attached to the 4-position of the 1,5-disubstituted pyrrolidine-2,3-dione core via a Schiff’ base (C=N) linkage in which the positive charge could be delocalized on both carbon and nitrogen atoms. In addition, even though there was an excess of water as compared to methylamine in the reaction mixture, the nucleophilicity of water molecules is lower than that of the aliphatic amine [22]. Hence, the transimination reaction [23] has occurred reversibly in which the imine (C=N) linkage-containing resonance forms **3a’**–**e’** will be attacked by methylamine (**4**) to yield the tetrahedral intermediate **6** and then, the intramolecular proton transfer will lead to intermediate **7**. It has been proven that Schiff’ bases show normally higher reactivity than the corresponding carbonyl compounds towards nitrogen-containing nucleophiles [24]. Therefore, it is reasonable that the transimination reaction between **3a’**–**e’** and **4** preferentially takes place at the carbon atom of the imine (C=N) linkage instead of the carbonyl carbons at the 2- or 3-positions. Lastly, the covalent bond between the tetrahedral carbon atom and the positively charged nitrogen atom is broken heterolytically, leading to the final pyrrolidine-2,3-dione derivatives **5a–e** and 4-methoxybenzylamine (Scheme 3). Due to the higher basicity of methylamine as compared to 4-methoxybenzylamine [25], the transimination equilibrium is shifted towards product **5a**–**e** and the weaker base, 4-methoxybenzylamine.

### X-ray study of 4-(1-methylamino)ethylidene-1,5-diphenylpyrrolidine-2,3-dione (**5a**)

**Figure 2:**
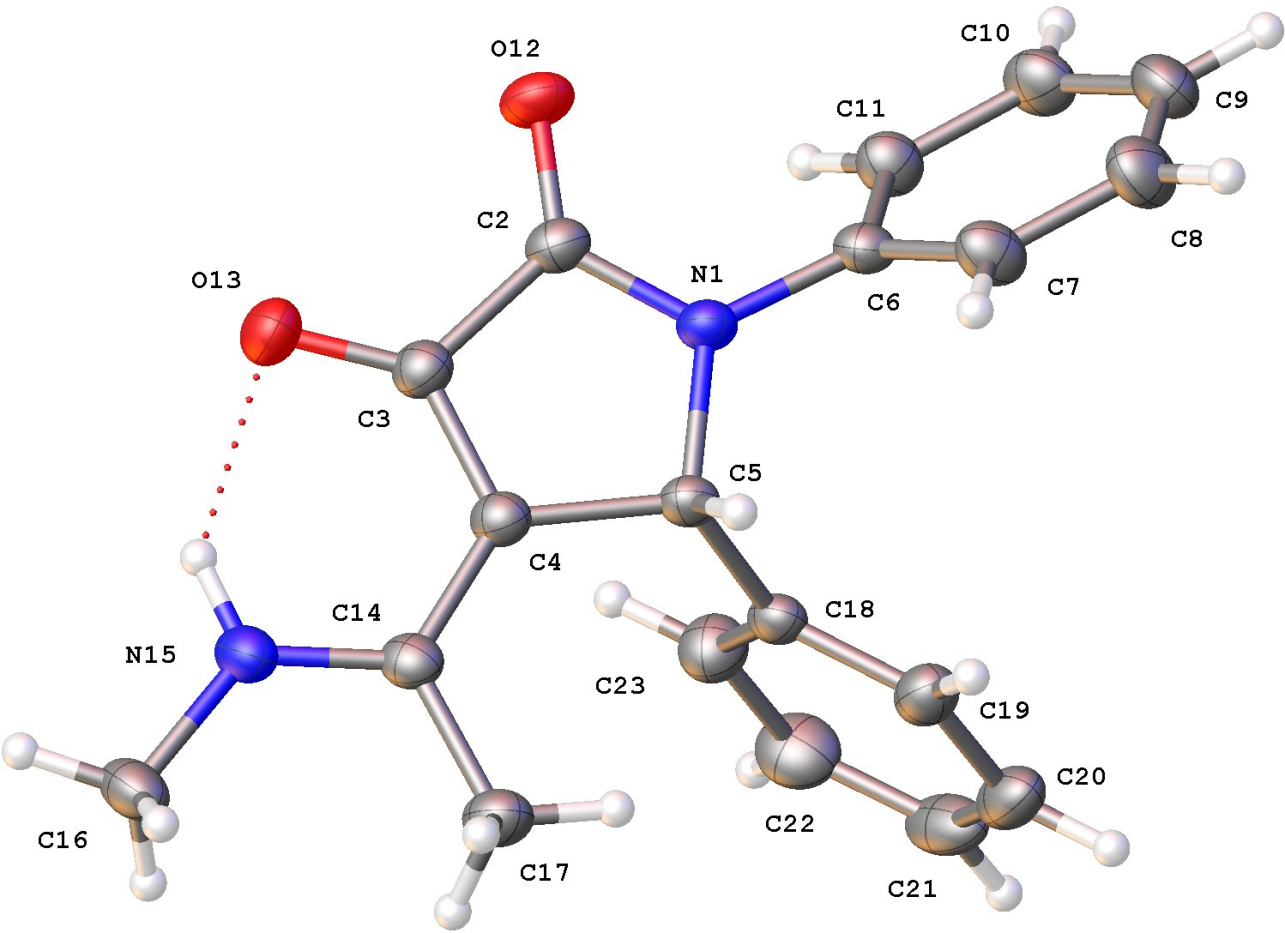
The molecular structure of **5a**, showing the atom-labelling scheme and displacement ellipsoids at the 30% probability level. The intramolecular hydrogen bond is shown as red dashed line.

Compound **5a** crystallizes in the triclinic space group *P−*1, with one molecule in the asymmetric unit (Figure 2). The pyrrolidine ring is planar (r.m.s. deviation = 0.004 Å) and forms a dihedral angle of 49.86(10) 86.22(11)° with the phenyl rings C6–C11 and C18–C23, respectively. The angle between both phenyl rings is 70.76(11)°. The stereochemistry around the double bond is *Z*, allowing an intramolecular N–H···O hydrogen bond between one of the carbonyl oxygen atoms and the amino group (Table S2 in Supporting Information File 1). The N and C atoms of the 1-methylamino)ethylidene group are coplanar with the pyrrolidine ring (max. deviation of 0.074(2) Å for atom C17).

The molecules form inversion dimers through N–H···O hydrogen bonding. Despite the presence of aromatic rings, no π–π stacking is observed in the crystal packing of the complexes, but only C–H···π interactions are present. Two C–H···O hydrogen bonds complete the interactions stabilizing the crystal packing (Figure S1 and Table S2 in Supporting Information File 1).

### Inhibitory activity of NO production

It is clear that overproduction of nitric oxide (NO) by the iNOS enzyme causes inflammation-related diseases. Moreover, large quantities of nitric oxide (NO) are released when RAW 264.7 cells are stimulated by lipopolysaccharides (LPS) [5,26]. Consequently, nitric oxide (NO) production in LPS-stimulated RAW264.7 macrophages was used to evaluate the anti-inflammatory capability of the five pyrrolidine-2,3-diones **5a–e** using dexamethasone as the reference drug (Table 2). Compounds **5c** and **5d** did not inhibit NO production in LPS-stimulated RAW264.7 macrophages while **5a**, **5b**, and **5e** exhibited inhibitory activity with IC_50_ values of 78.65 ± 6.88 µM, 95.66 ± 9.93 µM, and 43.69 ± 5.26 µM, respectively. Among the synthesized compounds **5a**–**e**, the best nitric oxide (NO) inhibitory capability was observed for compound **5e** (IC_50_ = 43.69 ± 5.26 µM) which is, however, still three times higher than that of the reference drug dexamethasone (IC_50_ = 13.25 ± 1.39 µM). At a concentration of 100 µM, the NO inhibition of pyrrolidine-2,3-dione derivatives **5a**, **5b**, and **5e** was 53.65%, 50.22%, and 63.65%, respectively, as compared to 85.04% of dexamethasone. It is clear that the presence of a nitro group or halogen atom on the benzene ring at the 5-position of pyrrolidine-2,3-dione core in compounds **5c** and **5d** could relate to the lower inhibitory activity of 23.76% and 38.41% at the concentration of 100 µM, respectively, as compared to **5a** and **5b**. However, the existence of a nitro group on the aromatic ring attached to the 1-position of that heterocyclic core in compound **5e** resulted in a dramatic increase in the NO inhibitory capability. More importantly, all studied compounds **5a–e** did not show cytotoxicity to macrophage cells; 90.35–95.74% cells survived at the concentration of 100 µM of **5a–e** (Table S3 in Supporting Information File 1).

**Table 2:** Inhibitory capability of pyrrolidine-2,3-diones **5a–e** on NO production.

Compound	IC_50_ (µM)

**5a**	78.65 ± 6.88
**5b**	95.66 ± 9.93
**5c**	>100
**5d**	>100
**5e**	43.69 ± 5.26
dexamethasone	13.25 ± 1.39

### Analysis of drug-likeness and ADMET characteristics

**Figure 3:**
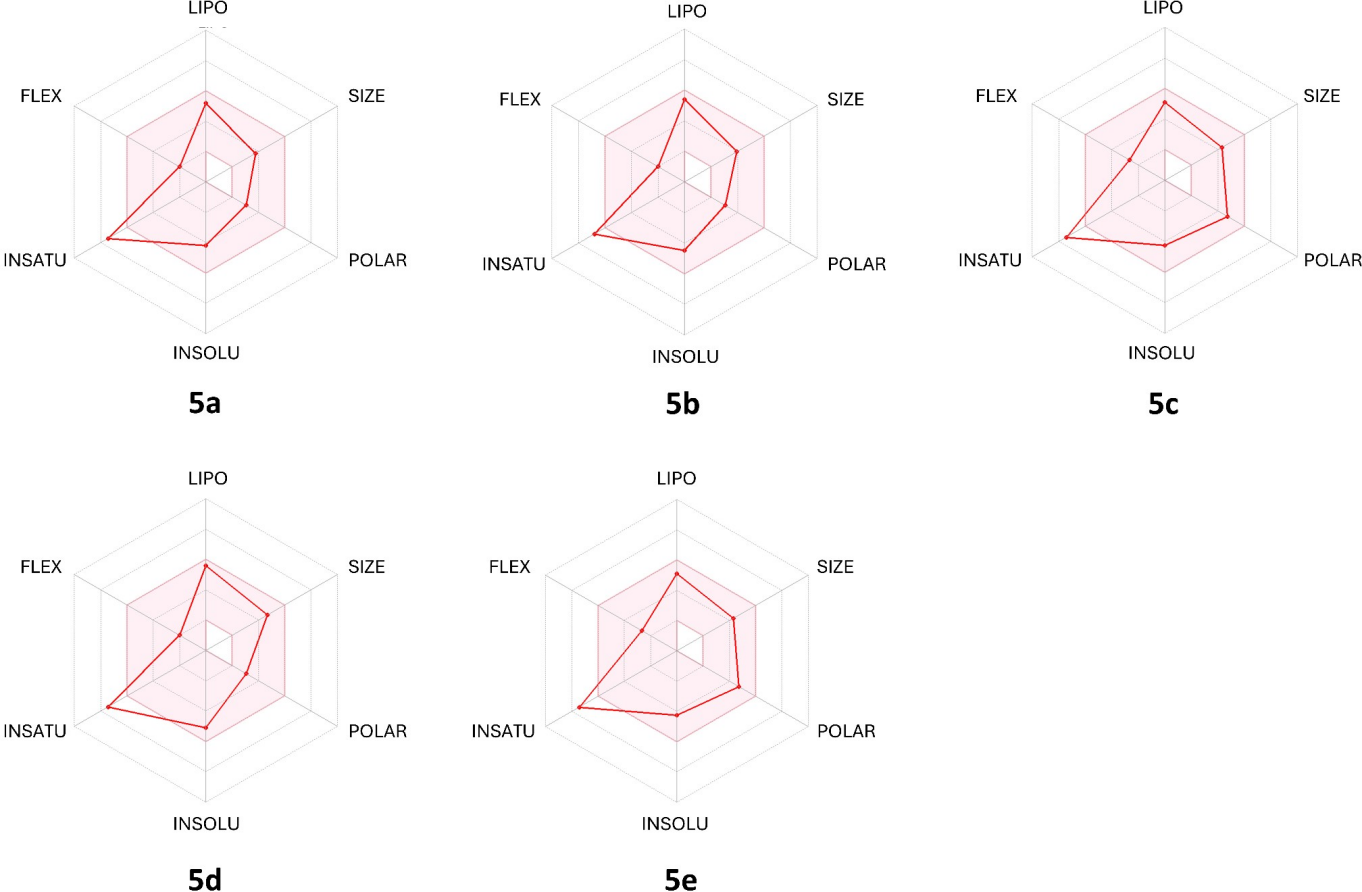
The bioavailability radar of studied compounds **5a–e**.

Drug-likeness serves as an essential preliminary evaluation in the drug discovery process. In this section, we investigate the drug-likeness profiles of the newly synthesized molecules by theoretically assessing whether they conform to established drug-likeness rules, including those of Lipinski, Veber, Ghose, Egan, and Muegge. A compound that violates multiple criteria is generally associated with poor oral bioavailability. As summarized in Table S4 of Supporting Information File 1, all potential candidates satisfy the drug-likeness criteria. In addition, the bioavailability radar (Figure 3) confirms that all compounds are predicted to exhibit favorable oral bioavailability, with two critical parameters, flexibility (FLEX) and polarity (POLAR) [27], aligning within the optimal range, as indicated by the pink area.

Following the prediction of drug-like properties, in silico evaluations of ADMET (absorption, distribution, metabolism, excretion, and toxicity) characteristics of all potential drug candidates were conducted using the pkCSM online tool [28]. The resulting data are presented in Table 3. The absorption capability of the examined molecules was assessed via two key parameters: Caco-2 membrane permeability and intestinal absorption. Caco-2 membrane permeability is widely recognized as a critical indicator of a compound's ability to permeate biological membranes, in which permeability is measured in log *P*_app_. A log *P*_app_ value greater than 0.9 is generally considered as the indicator of high permeability [28]. The analysis revealed that all five potential drugs demonstrated high permeability across the Caco-2 membrane, with log *P*_app_ values ranging from 1.082 to 1.472. Furthermore, intestinal absorption is classified as excellent when it falls within the range of 70% to 100% [29]. The data in Table 3 indicate that all studied compounds exhibit substantial absorption within the human intestines.

**Table 3:** ADMET properties of synthetic compounds **5a–e**.

	**5a**	**5b**	**5c**	**5d**	**5e**

Absorption					

Caco-2 permeability (log *P*_app_)	1.360	1.358	1.472	1.082	1.475
intestinal absorption (human) (%)	96.709	96.684	90.093	95.159	90.294

Distribution					

VDss (human) (log L/kg)	0.088	0.162	−0.185	0.139	−0.183
BBB permeability (log BB)	0.22	0.204	−0.62	0.189	−0.622
CNS permeability (log PS)	−2.143	−2.070	−2.343	−2.007	−2.356

Metabolism					

CYP2D6 substrate (yes/no)	no	no	no	no	no
CYP3A4 substrate (yes/no)	yes	yes	yes	yes	yes
CYP2D6 inhibitor (yes/no)	no	no	no	no	no
CYP3A4 inhibitor (yes/no)	no	no	no	no	no

Excretion					

total clearance (log mL/min/kg)	0.304	0.306	0.322	−0.077	0.274
renal OCT2 substrate (yes/no)	no	no	no	no	no

Toxicity					

AMES toxicity (yes/no)	no	no	yes	no	yes
skin sensitization	no	no	no	no	no

In terms of distribution, key factors influencing the distribution of the compounds comprise the volume of distribution at steady-state (VDss), blood–brain barrier (BBB) permeability, and central nervous system (CNS) penetration. VDss is considered elevated if the log VDss exceeds 0.45 [30]. BBB permeability is evaluated high when the log BB surpasses 0.3, while a log BB lower than −1 is indicative of low permeability [31]. CNS penetration is generally inferred when the log PS value is greater than −2, whereas values below −3 suggest an inability to penetrate the CNS [28]. The results presented in Table 3 display that all synthetic compounds possess low to moderate permeability across these barriers.

Metabolism was evaluated focusing on the cytochrome P450 enzyme system, particularly the CYP3A4 and CYP2D6 isoforms, which are responsible for metabolizing over 75% of the examined compounds [32]. Inhibition of these enzymes can result in elevated drug concentrations within the bloodstream due to reduced metabolism. The data in Table 3 indicate that none of the candidates acted as substrates or inhibitors of CYP2D6, even though all were substrates for CYP3A4, suggesting potential metabolism via this pathway without any inhibitory effects.

Excretion was assessed by examining total clearance, which is considered high when the log (mL/min/kg) exceeds 0.7, intermediate when that value ranges from 0.3 to 0.7, and low when it is below 0.3 [33]. The total clearance of the studied compounds was observed to range from −0.077 to 0.322 mL/min/kg, indicating low to moderate clearance rates. Additionally, renal excretion via the organic cation transporter 2 (OCT2) was examined, with OCT2 playing a pivotal role in the renal elimination of both xenobiotics and endogenous compounds. The predicted results suggest that none of the studied compounds act as OCT2 substrates.

Toxicity was assessed using the Ames test, a widely accepted method for evaluating the mutagenic potential of compounds through bacterial assays. The Ames test results indicated that compounds **5c** and **5e** exhibited toxicity. Moreover, none of the tested molecules demonstrated potential for skin sensitization. Consequently, further experimental investigations are necessary to elucidate the toxicological profiles of compounds **5c** and **5e** prior to consideration for drug development.

### Analysis of reactivity descriptors

The primary reactivity descriptors, such as frontier molecular orbital energies (*E*_HOMO_ and *E*_LUMO_), energy gap (Δ*E*_L-H_), ionization energy (IE), electron affinity (EA), electronegativity (χ), hardness (η), and softness (S), were determined for the studied compounds and are summarized in Table 4. A detailed comparison of these parameters highlights significant differences in the chemical stability and reactivity of the compounds, reflecting their unique electronic characteristics and potential functional roles.

**Table 4:** The chemical reactivity characteristics of compounds **5a–e**.

Compound	*E*_HOMO_ (eV)	*E*_LUMO_ (eV)	Δ*E*_L-H_ (eV)	IE (eV)	EA (eV)	χ (eV)	η (eV)	S (1/eV)

**5a**	−6.088	−1.967	4.121	6.088	1.967	4.027	2.061	0.485
**5b**	−6.040	−1.935	4.104	6.040	1.935	3.987	2.052	0.487
**5c**	−6.429	−3.032	3.397	6.429	3.032	4.731	1.699	0.589
**5d**	−6.205	−2.073	4.132	6.205	2.073	4.139	2.066	0.484
**5e**	−6.389	−2.694	3.696	6.389	2.694	4.542	1.848	0.541

As reported in Table 4, compound **5c** demonstrates the highest reactivity and lowest stability, as evidenced by its smallest Δ*E*_L-H_ (3.397 eV), highest softness (0.589), and lowest hardness (1.699 eV). Conversely, compound **5d** is the most stable, with the largest Δ*E*_L-H_ (4.132 eV) and the lowest softness (0.484), indicative of minimal reactivity. Compounds **5a** and **5b** exhibit moderate stability and reactivity, sharing similar Δ*E*_L-H_ values (≈4.1 eV) and softness values (0.485 and 0.487, respectively). Compound **5e** keeps a balance stability and reactivity with a Δ*E*_L-H_ of 3.696 eV and a softness value of 0.541, suggesting a relatively higher reactivity than **5a**, **5b**, and **5d**. The result analysis reveals that the reactivity increases in the order: **5d** < **5a** ≈ **5b** < **5e** < **5c**, while stability decreases in the same sequence. Additionally, high EA values for **5c** (3.032 eV) and **5e** (2.694 eV) emphasize their strong electron-accepting capabilities, while the high IE of **5c** (6.429 eV) indicates its resistance to electron donation. These findings suggest that compounds **5c** and **5e** are highly reactive and suitable for applications requiring active electron transfer, whereas **5d**, followed by **5a** and **5b**, exhibits greater stability which are more appropriate for interactions of low chemical reactivity.

### Analysis of molecular docking

**Figure 4:**
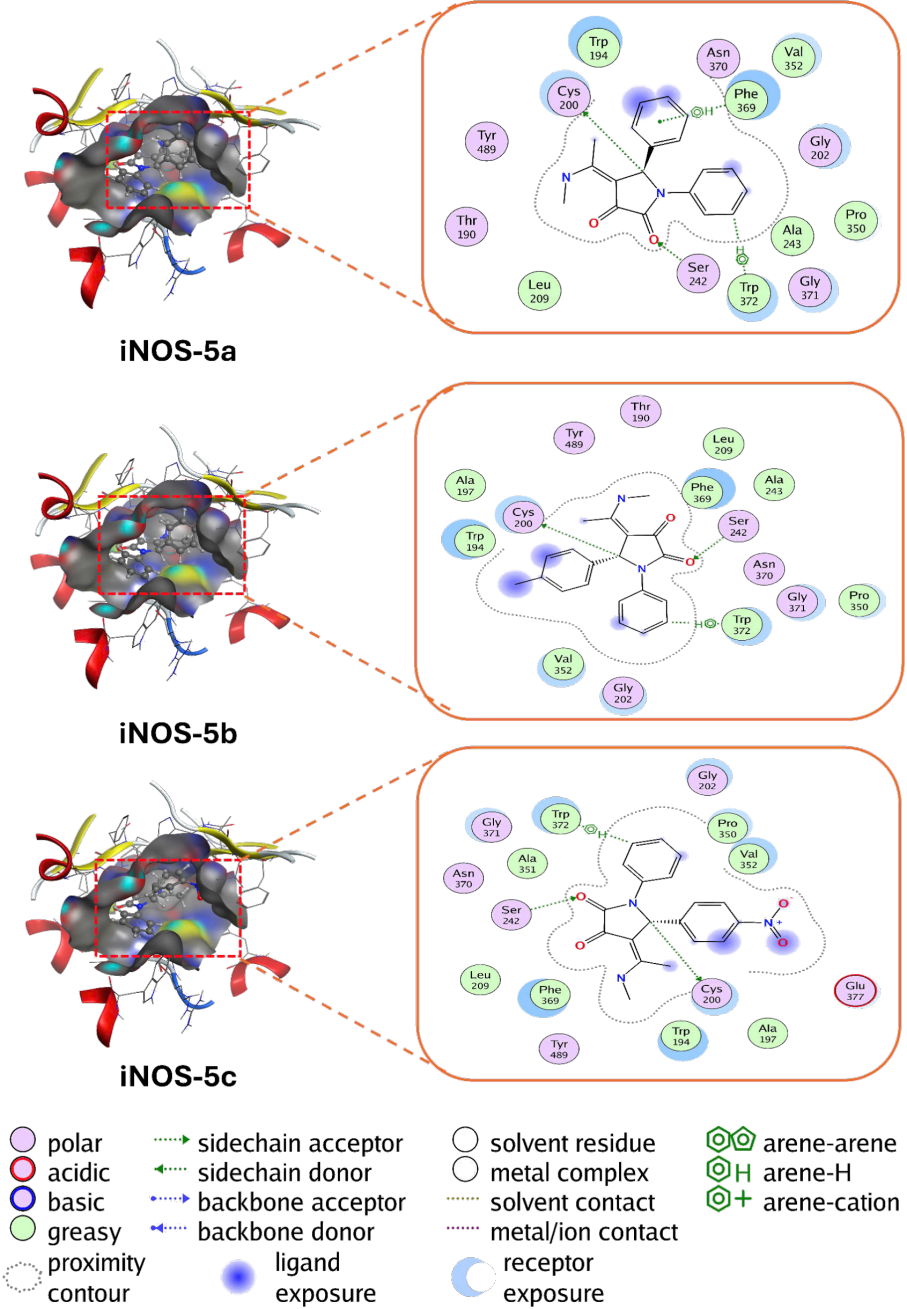
The interactions of potential drugs **5a**–**c** in the active site of enzyme iNOS.

**Figure 5:**
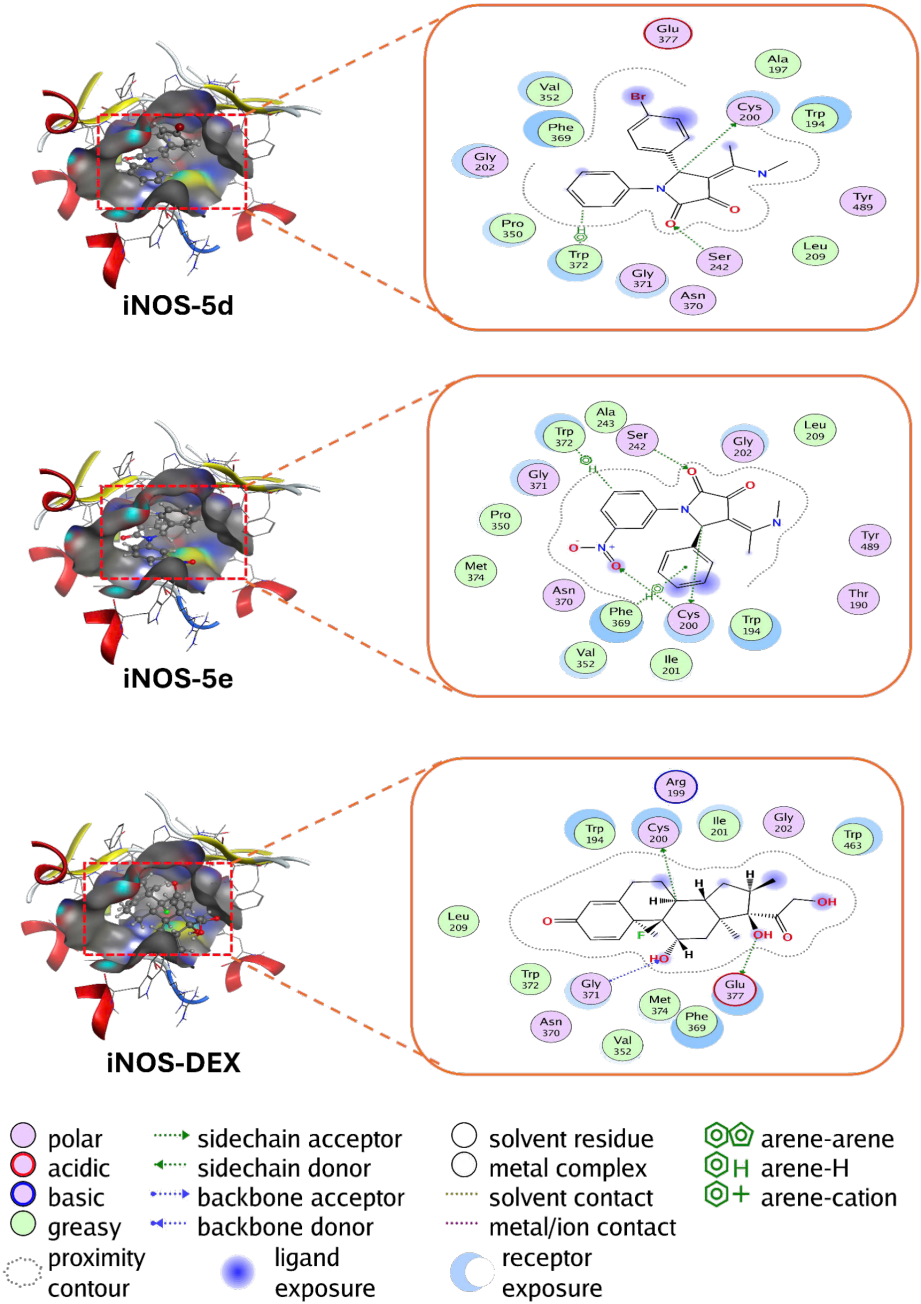
The interactions of potential drugs **5d** and **5e** and control drug (DEX) in the active site of enzyme iNOS.

In this section, molecular docking studies were conducted using iNOS as the target enzyme, with all five synthetic compounds serving as ligands (Figure 4 and Figure 5). Additionally, dexamethasone (DEX) was employed as an experimental control for comparative purposes [34–36]. The docking scores (DS), reported in Table 5 as binding affinities, reveal that negative DS values correspond to stronger binding affinities between the ligand and protein which enhance the stability of the complex [37–38].

**Table 5:** Molecular docking results of potential drugs with enzyme iNOS.

ligand–protein complex	DS (kcal/mol)	hydrogen bonds	van der Waals interactions

**iNOS–5a**	−8.94	Cys200, Ser242	Thr190, Trp194, Gly202, Leu209, Ala243, Gly371, Pro350, Val352, Phe369, Asn370, Trp372, Tyr489
**iNOS–5b**	−9.14	Cys200, Ser242	Thr190, Trp194, Ala197, Gly202, Leu209, Ala243, Pro350, Val352, Phe369, Asn370, Gly371, Tyr489
**iNOS–5c**	−9.26	Cys200, Ser242	Trp194, Ala197, Gly202, Pro350, Val352, Leu209, Ala351, Phe369, Asn370, Gly371, Trp372, Glu377, Tyr489
**iNOS–5d**	−9.14	Cys200, Ser242	Trp194, Ala197, Gly202, Leu209, Pro350, Val352, Phe369, Asn370, Gly371, Trp372, Glu377, Tyr489
**iNOS–5e**	−9.51	Cys200, Ser242	Thr190, Trp194, Ile201, Gly202, Leu209, Ala243, Pro350, Val352, Phe369, Asn370, Gly371, Trp372, Met374, Tyr489
**iNOS–DEX**	−8.55	Cys200, Gly371, Glu377	Trp194, Arg199, Ile201, Gly202, Leu209, Val352, Phe369, Asn370, Trp372, Met374, Trp463

The molecular docking study evaluated the binding affinities of potential drug candidates (**iNOS–5a**, **5b**, **5c**, **5d**, **5e**) and the reference compound (**iNOS–DEX**) with the inducible nitric oxide synthase (iNOS) enzyme (Figure 4 and Figure 5). The docking scores (DS) revealed a range of binding affinities, with values ranging from −8.55 kcal/mol (**iNOS–DEX**) to −9.51 kcal/mol (**iNOS–5e**). Notably, **iNOS–5e** exhibited the most favorable binding energy, suggesting it has the strongest interaction with the iNOS enzyme. Hydrogen bonding analysis demonstrated that all ligands consistently interacted with Cys200 and Ser242, key residues in the enzyme's active site, underscoring their critical role in ligand stabilization. In addition to hydrogen bonding, extensive van der Waals interactions were observed, particularly involving residues such as Thr190, Trp194, Gly202, Pro350, Phe369, and Tyr489, further contributing to the stabilization of the ligand–protein complexes. More importantly, the occurrence of an electron-withdrawing group, nitro group (NO_2_), on the aromatic ring linked to the 1-position of the pyrrolidine-2,3-dione core may help compound **5e** enhance its interaction with iNOS via the optimization of the hydrogen bond with Cys200. These results highlight the potential of compound **5e** as a lead compound for further development in targeting iNOS-related pathologies.

Experimental results have confirmed that compound **5e** is a promising candidate for inhibiting the enzyme nitric oxide synthase (iNOS). Specifically, **iNOS–5e** demonstrated significant inhibition of nitric oxide (NO) production, with an IC_50_ value of 43.69 ± 5.26 µM, the lowest among the designed compounds. This result highlights its substantial efficacy in suppressing iNOS activity, outperforming other compounds in the group, such as **5a** (IC_50_ = 78.65 ± 6.88 µM) and **5b** (IC_50_ = 95.66 ± 9.93 µM). These findings suggest that pyrrolidine-2,3-dione derivative **5e** holds significant potential as an effective iNOS inhibitor.

## Conclusion

In this study, a series of 4-(1-methylamino)ethylidene-1,5-disubstituted pyrrolidine-2,3-diones were successfully synthesized via reversible transimination reaction. The structure of all compounds was evaluated using nuclear magnetic resonance (NMR) spectroscopy and, especially, with the molecular structure of compound **5a** further confirmed through single-crystal X-ray diffraction analysis. The X-ray study provided precise details about the molecular geometry, validating the predicted configurations and ensuring the reliability of the synthesized structures.

Reactivity descriptors, including Δ*E*_L-H_, softness, and hardness, were analyzed to evaluate the stability and reactivity of compounds. This trend aligns with the increasing reactivity order: **5d** < **5a** ≈ **5b** < **5e** < **5c**. Molecular docking simulations provided insights into the binding interactions of these compounds with the inducible nitric oxide synthase (iNOS) enzyme. Compound **5e** exhibited the strongest binding affinity, with a docking score of −9.51 kcal/mol, outperforming the reference compound dexamethasone (**iNOS–DEX**, −8.55 kcal/mol). Key hydrogen bonds with residues Cys200 and Ser242, together with extensive van der Waals interactions involving Thr190, Trp194, Gly202, Pro350, Phe369, and Tyr489, further stabilized the ligand–protein complexes. Experimental evaluations have confirmed these findings in which compound **5e** demonstrated the most potent inhibitory activity against iNOS, achieving an IC_50_ value of 43.69 ± 5.26 µM substantially lower than that of **5a** (IC_50_ = 78.65 ± 6.88 µM) and **5b** (IC_50_ = 95.66 ± 9.93 µM).

The integration of synthetic approaches, structural studies, computational analyses, and experimental validations underscores the potential of 4-(1-methylamino)ethylidene-1,5-disubstituted pyrrolidine-2,3-diones, particularly compound **5e**, as effective candidates for anti-inflammatory drug development targeting iNOS-related pathologies. Future research should prioritize extensive in vivo studies and clinical evaluations to further explore their therapeutic potential and safety profiles.

## Experimental

### Experimental methods

All chemicals were purchased from Merck, Sigma-Aldrich and Acros without further purification. For column chromatography, 70–230 mesh silica 60 (E. M. Merck) was used as the stationary phase. Electrospray ionization–high-resolution mass spectra (ESI–HRMS) were acquired on a SCIEX X500 QTOF instrument in the positive ion mode. A Büchi melting point B-545 apparatus was used to determine the melting points of all products. All NMR spectra were recorded on a Bruker Avance II+ 600 MHz instrument and chemical shifts (δ) are reported in ppm (parts per million) relative to tetramethylsilane (TMS) or internal deuterated solvent signals.

### Computational methods

**Prediction of drug-likeness and ADMET properties**: Drug-likeness and ADMET (absorption, distribution, metabolism, excretion, and toxicity) analyses are critical tools for assessing the pharmacokinetic profile of a chemical compound prior to its selection as a therapeutic candidate. Screening criteria were applied based on established guidelines, including Muegge’s rule (Bayer) [39], Ghose’s rule [40], Egan’s rule (Pharmacia) [41], Veber’s rule (GSK) [42], and Lipinski’s rule of five [43]. Additionally, bioavailability radar assessments were performed using the SwissADME online tool [44]. ADMET studies were further conducted using the pkCSM web server [28].

**Docking molecular simulation**: The structure of nitric oxide synthase (iNOS) protein (PDB ID: 3E7G) [45] was obtained from the Protein Data Bank. Protonation of the protein was carried out using the Protonate 3D tool in MOE to assign correct protonation states at pH 7.4. The docking algorithm used was the Triangle Matcher placement method, which generates initial poses by aligning ligand atoms to complementary receptor atoms. Molecular docking simulations were performed using the MOE software suite [46] to evaluate the binding activities of the newly synthesized compounds and the reference drug dexamethasone (DEX) against the iNOS enzyme. The interactions between the ligands and the target enzyme were further visualized and analyzed using Discovery Studio software [47].

**Density functional theory (DFT) method**: The molecular structures of the tested compounds were optimized at the M062X/6-31+G(d) level of theory using Gaussian 16 software [48]. The reactivity, stability, and electronic properties of molecules determined by key parameters such as frontier molecular orbital energies (*E*_HOMO_ and *E*_LUMO_), energy gap (Δ*E*_L-H_), ionization energy (IE), electron affinity (EA), electronegativity (χ), hardness (η), and softness (S). The calculations were based on established formulas, as described in literature [49].

## Supporting Information

File 1 Synthetic procedures, compound characterization, X-ray crystallographic data, bioassay for NO inhibition, NMR and ESI-HRMS spectra for all reported compounds.

## Data Availability

All data that supports the findings of this study is available in the published article and/or the supporting information of this article.
