# Supplementary material for: 4-(1-Methylamino)ethylidene-1,5-disubstituted pyrrolidine-2,3-diones: synthesis, anti-inflammatory effect and in silico approaches
Source: Beilstein J Org Chem. 2025 Apr 24;21:817–29. doi: 10.3762/bjoc.21.65 (PMC12035876; doi:10.3762/bjoc.21.65)
Supplement: File 1 — Synthetic procedures, compound characterization, X-ray crystallographic data, bioassay for NO inhibition, NMR and ESI-HRMS spectra for all reported compounds. [file Beilstein_J_Org_Chem-21-817-s001.pdf]

BEILSTEIN JOURNAL OF ORGANIC CHEMISTRY

Supporting Information

for

4-(1-Methylamino)ethylidene-1,5-disubstituted pyrrolidine-2,3-diones: synthesis, anti-inflammatory effect and in silico approaches

Nguyen Tran Nguyen, Vo Viet Dai, Luc Van Meervelt, Do Thi Thao and Nguyen Minh Thong

Beilstein J. Org. Chem. **2025,** *21,* 817–829. doi:10.3762/bjoc.21.65

Synthetic procedures, compound characterization, X-ray crystallographic data, bioassay for NO inhibition, NMR and ESI-HRMS spectra for all reported compounds

License and Terms: This is a supporting information file under the terms of the Creative Commons Attribution License (https://creativecommons.org/licenses/by/4.0). Please note that the reuse, redistribution and reproduction in particular requires that the author(s) and source are credited and that individual graphics may be subject to special legal provisions.
The license is subject to the *Beilstein Journal of Organic Chemistry* terms and conditions: (https://www.beilstein-journals.org/bjoc/terms)

Table of contents

1. Materials, methods and spectra data.. S1

2. Determination of crystal structure of **5a**.. S7

3. Bioassay for NO inhibition.. S11

4. ADMET properties... S14

5. References.. S17

6. Spectra.. S18

1. Materials, methods and spectra data

Spectra data of compounds **1a**, **1b**, **1d**, **1e**, **3a**, **3b**, **3d** and **3e** have been described in the literature [1-3].

General procedure for the synthesis of 4-acetyl-3-hydroxy-3-pyrroline-2-one (1a–e) in a manner similar to reference [1] A mixture of aromatic aldehyde (1.5 equiv, 0.75 mmol), aromatic amine (1.0 equiv, 0.5 mmol) and glacial acetic acid (1.0 mL) was added to a 5 mL round-bottomed flask equipped with a mechanical stirrer. The solution was stirred vigorously under argon (Ar) atmosphere at room temperature for 1 hour to yield the imine intermediate. Consecutively, ethyl 2,4-dioxovalerate (1.0 equiv, 0.5 mmol) was added to the flask and the resulting mixture was stirred vigorously at room temperature under Ar atmosphere for 2–4 hours. To obtain pure products, crude products were purified via recrystallization [1].

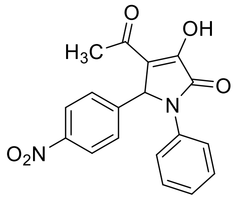

Synthesis of 4-acetyl-3-hydroxy-1-phenyl-5-(4-nitrophenyl)-3-pyrroline-2-one (1c) in a manner similar to reference [1]: 4-nitrobenzaldehyde (113.3 mg, 1.5 equiv, 0.75 mmol), aniline (0.046 mL, 1.0 equiv, 0.5 mmol), ethyl 2,4-dioxovalerate (0.07 mL, 1.0 equiv, 0.5 mmol) and glacial acetic acid (1.0 mL), under Ar atmosphere. Reaction time is 4 h. The crude product was purified via recrystallization in a solvent mixture of CH_2Cl_2 and toluene to obtain **1c** (113.4 mg, 67% yield) as an off white solid. m.p. 198-199 ˚C.

^{1}H NMR (600 MHz, $CDCl_3$) δ 8.11 (d, ^3J(H,H) = 8.79 Hz, 2H; Ar-H), 7.44 (d, ^3J(H,H) = 8.75 Hz, 2H; Ar-H), 7.43 (dd, ^4J(H,H) = 1.18 Hz ,^3J(H,H) = 8.74 Hz, 2H; Ar-H), 7.32-7.29 (m, 2H; Ar-H), 7.16 (t, ^3J(H,H) = 7.44 Hz, 1H; Ar-H), 5.93 (s, 1H), 2.36 ppm (s, 3H; CH_3). **^{13}C NMR (**150 MHz, DMSO-d6) δ 164.50, 136.57, 136.00, 131.01, 129.91, 128.86, 128.72, 128.17, 125.50, 125.28, 122.47, 120.72, 59.77 ppm. **^{13}C NMR (**150 MHz, $CDCl_3$) δ 164.46, 148.16, 142.92, 135.48, 129.56, 128.78, 126.96, 124.18, 122.38, 119.92, 111.32, 61.67, 29.79 ppm. **HRMS** (ESI-TOF MS/MS) *m/z* $[M + H]^+$ calcd for $C_{18}H_{14}N_2O_5$: 339.0981; found: 339.0970

General procedure for the synthesis of 4-[1-(4-methoxybenzyl)amino]ethylidene-1,5-disubstituted pyrrolidine-2,3-dione (3a–e) in a manner similar to reference [1] 4-acetyl-3-hydroxy-3-pyrroline-2-one (1.0 equiv), 4-methoxybenzylamine (4.0 equiv) and absolute ethanol (0.34 mL) were added to a screw-capped reaction tube equipped with a magnetic stirring bar. The reaction mixture was stirred vigorously at 80 °C for 5 hours and then, hexane (5 mL) was added to the reaction tube. The resulting precipitate was purified via column chromatography (eluent CH_2Cl_2/CH_3OH) to obtain pure product [1].

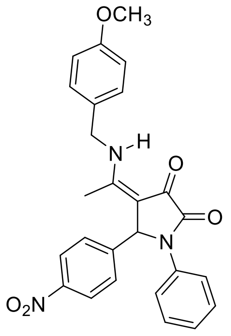

Synthesis of 4-[1-(4-methoxybenzyl)amino]ethylidene-1-phenyl-5-(4-nitrophenyl)pyrrolidine-2,3-dione (3c) in a manner similar to reference [1]. A mixture of 4-acetyl-3-hydroxy-1-phenyl-5-(4-nitrophenyl)-3-pyrroline-2-one (**1c**, 50.0 mg, 1.0 equiv, 0.148 mmol), 4-methoxybenzylamine (**2**, 0.077 mL, 4.0 equiv, 0.592 mmol) and ethanol (0.34 mL) was stirred vigorously at 80 °C for 5 h. The crude product was purified by column chromatography (silica gel, eluent CH_2Cl_2/CH_3OH 5:0.06) to yield **3c** (60.1 mg, 88% yield) as a light yellow solid. m.p. 226-227 ˚C.

^{1}H NMR (600 MHz, $CDCl_3$) δ 11.64 (s_{br}, 1H; NH), 8.07 (d, ^3J(H,H) = 8.70 Hz, 2H; Ar-H), 7.38 (d, ^3J(H,H) = 8.28 Hz, 2H; Ar-H), 7.36 (d, ^3J(H,H) = 7.84 Hz, 2H; Ar-H), 7.27-7.25 (m, 2H; Ar-H), 7.12-7.10 (m, 3H; Ar-H), 6.86 (d, ^3J(H,H) = 8.65 Hz, 2H; Ar-H), 5.85 (s, 1H), 4.41 (dd, ^2J(H,H) = 6.14 Hz, ^3J(H,H) = 15.52 Hz, 1H; CH_2), 4.30 (dd, ^2J(H,H) = 6.14 Hz, ^3J(H,H) = 15.52 Hz, 1H; CH_2), 3.79 (s, 3H; -OCH_3), 1.82 ppm (s, 3H; CH_3). **^{13}C NMR (**150 MHz, $CDCl_3$) δ 164.01, 159.62, 147.93, 146.50, 136.31, 129.25, 128.97, 128.36, 127.74, 126.75, 124.23, 123.81, 114.68, 60.65, 55.49, 46.99, 15.86 ppm. **HRMS** (ESI-TOF MS/MS) *m/z* $[M + H]^+$ calcd for $C_{26}H_{23}N_3O_5$: 458.1716; found: 458.1690

Procedure for the synthesis of 4-(1-methylamino)ethylidene-1,5-diphenylpyrrolidine-2,3-dione (5a) using absolute ethanol as solvent: To a screw-capped reaction tube equipped with a magnetic stirring bar, 4-[1-(4-methoxybenzyl)amino]ethylidene-1,5-diphenylpyrrolidine-2,3-dione (30.0 mg, 0.073 mmol, 1.0 equiv,), methylamine (40% in water, 0.025 mL, 0.292 mmol, 4.0 equiv) and absolute ethanol (0.3 mL) were added. The reaction mixture was stirred vigorously at 80 °C for 2 hours in the manner similar to reference [1] for the synthesis of **5a** from 4-acetyl-3-hydoxy-1,5-diphenyl-3-pyrrolin-2-one (1.0 equiv) and methylamine (40% in water, 4.0 equiv) in absolute ethanol. The progress of the reaction was monitored by thin-layer chromatography (TLC; eluent CH_2Cl_2/CH_3OH 5:0.1). After the reaction was complete, distilled water was added to the reaction tube and the mixture was stirred for 15 minutes at room temperature. The resulting precipitate was filtered and purified via column chromatography (silicagel, eluent CH_2Cl_2/CH_3OH) to yield pure product **5a** (18.05 mg, 80.8%) as a white solid.

General procedure for the synthesis of 4-(1-methylamino)ethylidene-1,5-disubstituted pyrrolidine-2,3-diones (5a–e) without using absolute ethanol: To a screw-capped reaction tube equipped with a magnetic stirring bar was added 4-[1-(4-methoxybenzyl)amino]ethylidene-1,5-disubstituted pyrrolidine-2,3-dione (1.0 equiv) and methylamine (40% in water) (47 equiv, 0.3 mL). The reaction mixture was stirred vigorously at 80 °C for 1–3 h and the reaction progress was monitored by TLC (eluent CH_2Cl_2/CH_3OH 5:0.1). After the reaction was complete, distilled water was added and the mixture was stirred at room temperature for 15 minutes. The precipitate was isolated by filtration and purified via column chromatography (silicagel, eluent CH_2Cl_2/CH_3OH) to yield the pure product.

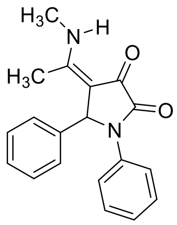

4-(1-Methylamino)ethylidene-1,5-diphenylpyrrolidine-2,3-dione (5a): the mixture of 4-[1-(4-methoxybenzyl)amino]ethylidene-1,5-diphenylpyrrolidine-2,3-dione (**3a**, 30.0 mg, 0.073 mmol, 1 equiv) and methylamine (40% in water, 0.3 mL, 3.431 mmol, 47 equiv) was stirred vigorously at 80

°C for 1 hour. The crude product was purified by column chromatography (silica gel, eluent CH_2Cl_2/CH_3OH 5:0.1) affording **5a** (20.6 mg, 92.2% yield) as a white solid. m.p. 284-290 °C.

^{1}H NMR (600 MHz, $CDCl_3$) δ 11.20 (d_{br}, ^3J(H,H) = 6.64 Hz, 1H; N−H), 7.43 (d, ^3J(H,H) = 8.22 Hz, 2H; Ar-H), 7.26 – 7.23 (m, 2H; Ar-H), 7.19 – 7.14 (m, 5H; Ar-H), 7.07 (t, ^3J(H,H) = 7.51 Hz, 1H; Ar-H), 5.73 (s, 1H), 2.76 (d, ^3J(H,H) = 5.23 Hz, 3H; CH_3), 1.78 ppm (s, 3H; CH_3). **^{13}C NMR** (150 MHz, $CDCl_3$) δ 176.55, 165.52, 164.62, 138.78, 136.88, 128.83, 128.75, 128.31, 128.14, 126.02, 123.99, 107.10, 61.31, 29.89, 15.34 ppm. **HRMS** (ESI-TOF MS/MS) m/z $[M + H]^+$ calcd for $C_{19}H_{18}N_2O_2$: 307.1447; found: 307.1430

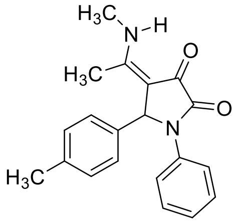

5-(4-Methylphenyl)-4-(1-methylamino)ethylidene-1-phenylpyrrolidine-2,3-dione (5b): the mixture of 5-(4-methylphenyl)-4-[1-(4-methoxybenzyl)amino]ethylidene-1-phenylpyrrolidine-2,3-dione (**3b**, 31.0 mg, 0.073 mmol, 1 equiv) and methylamine (40% in water, 0.3 mL, 3.431 mmol, 47 equiv) was stirred vigorously at 80 °C for 1 hour. The crude product was purified by column chromatography (silica gel, eluent CH_2Cl_2/CH_3OH 5:0.06) affording **5b** (20.8 mg, 89.4% yield) as a white solid. m.p. 272-274 °C.

^{1}H NMR (600 MHz, $CDCl_3$) δ 11.19 (d_{br}, ^3J(H,H) = 5.64 Hz, 1H; N−H), 7.43 (d, ^3J(H,H) = 7.20 Hz, 2H; Ar-H), 7.25 (d, ^3J(H,H) = 8.55 Hz, 2H; Ar-H), 7.08 – 7.05 (m, 3H; Ar-H), 6.98 (d, ^3J(H,H) = 7.84 Hz, 2H; Ar-H), 5.70 (s, 1H), 2.75 (d, ^3J(H,H) = 5.14 Hz, 3H; CH_3), 2.21 (s, 3H; CH_3), 1.78 ppm (s, 3H; CH_3). **^{13}C NMR** (150 MHz, $CDCl_3$) δ 176.52, 165.52, 164.63, 138.00, 136.94, 135.63, 129.43, 128.78, 128.00, 125.90, 123.92, 107.17, 60.97, 29.84, 21.16, 15.32 ppm. **HRMS** (ESI-TOF MS/MS) *m/z* $[M + H]^+$ calcd for $C_{20}H_{20}N_2O_2$: 321.1603; found: 321.1588

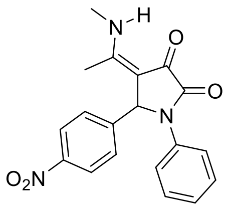

4-(1-Methylamino)ethylidene-1-phenyl-5-(4-nitrophenyl)pyrrolidine-2,3-dione (5c): the mixture of 4-[1-(4-methoxybenzyl)amino]ethylidene-1-phenyl-5-(4-nitrophenyl)pyrrolidine-2,3-dione (**3c**, 33.3 mg, 0.073 mmol, 1 equiv) and methylamine (40% in water, 0.3 mL, 3.431 mmol, 47 equiv) was stirred vigorously at 80 °C for 1 hour. The crude product was purified by column chromatography (silica gel, eluent CH_2Cl_2/CH_3OH 5:0.08) affording **5c** (23.0 mg, 90.1% yield) as a white solid. m.p. 264-265 ˚C.

^{1}H NMR (600 MHz, $CDCl_3$) δ 11.13 (d_{br}, 1H; NH), 8.05 (d, ^3J(H,H) = 8.77 Hz, 2H; Ar-H), 7.47 (dd, ^4J(H,H) = 1.18 Hz ,^3J(H,H) = 8.71 Hz, 2H; Ar-H), 7.38 (d, ^3J(H,H) = 8.79 Hz, 2H; Ar-H), 7.30-7.27 (m, 2H; Ar-H), 7.11 (t, ^3J(H,H) = 7.43 Hz, 1H; Ar-H), 5.90 (s, 1H), 2.64 (d, ^3J(H,H) = 5.34 Hz, 3H; CH_3-NH-), 1.78 ppm (s, 3H; CH_3). **^{13}C NMR (**150 MHz, $CDCl_3$) δ 176.14, 165.77, 164.26, 147.82, 146.53, 136.33, 129.17, 129.03, 126.43, 124.11, 123.71, 106.23, 60.20, 29.86, 15.66 ppm. **HRMS** (ESI-TOF MS/MS) *m/z* $[M + H]^+$ calcd for $C_{19}H_{17}N_3O_4$: 352.1297; found: 352.1290

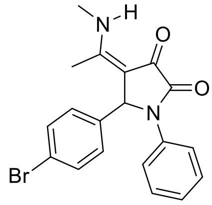

4-(1-Methylamino)ethylidene-1-phenyl-5-(4-bromophenyl)pyrrolidine-2,3-dione (5d): the mixture of 4-[1-(4-methoxybenzyl)amino]ethylidene-1-phenyl-5-(4-bromophenyl)pyrrolidine-2,3-dione (**3d**, 35.8 mg, 0.073 mmol, 1 equiv) and methylamine (40% in water, 0.3 mL, 3.431 mmol, 47 equiv) was stirred vigorously at 80 °C for 1 hour. The crude product was purified by column chromatography (silica gel, eluent CH_2Cl_2/CH_3OH 5:0.06) affording **5d** (23.9 mg, 85.3% yield) as a white solid. m.p. 289-290 ˚C.

^{1}H NMR (600 MHz, $CDCl_3$) δ 11.17 (d_{br}, 1H; NH), 7.42 (dd, ^4J(H,H) = 1.38 Hz, ^3J(H,H) = 8.31 Hz, 2H; Ar-H), 7.32 (d, ^3J(H,H) = 8.41 Hz, 2H; Ar-H), 7.28 (d, ^3J(H,H) = 7.87 Hz, 2H; Ar-H), 7.10 (t, ^3J(H,H) = 7.41 Hz, 1H; Ar-H), 7.07 (d, ^3J(H,H) = 8.44 Hz, 2H; Ar-H), 5.72 (s, 1H), 2.72 (d, ^3J(H,H) = 5.14 Hz,

3H; CH_3-NH-), 1.77 ppm (s, 3H; CH_3). **^{13}C NMR (**150 MHz, $CDCl_3$) δ 176.37, 165.61, 164.45, 138.00, 136.59, 132.01, 129.79, 129.00, 126.23, 123.93, 122.27, 106.66, 60.57, 29.88, 15.47 ppm. **HRMS** (ESI-TOF MS/MS) *m/z* $[M + H]^+$ calcd for $C_{19}H_{17}BrN_2O_2$: 385.0552; found: 385.0530

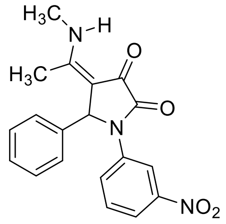

4-(1-Methylamino)ethylidene-5-phenyl-1-(3-nitrophenyl)pyrrolidine-2,3-dione (5e): the mixture of 4-[1-(4-methoxybenzyl)amino]ethylidene-5-phenyl-1-(3-nitrophenyl)pyrrolidine-2,3-dione (**3e**, 33.3 mg, 0.073 mmol, 1 equiv) and methylamine (40% in water, 0.3 mL, 3.431 mmol, 47 equiv) was stirred vigorously at 80 °C for 1 hour. The crude product was purified by column chromatography (silica gel, eluent CH_2Cl_2/CH_3OH 5:0.1) affording **5e** (22.6 mg, 88.3% yield) as a white solid. m.p. 250-251 ˚C. **^{1}H NMR** (600 MHz, $CDCl_3$) δ 11.42 (s_{br}, 1H; NH), 8.25 (t, ^4J(H,H) = 2.16 Hz, 1H; Ar-H), 8.05 (ddd, ^3J(H,H) = 8.19 Hz, ^4J(H,H) = 2.11 Hz, ^4J(H,H) = 2.16 Hz, 1H; Ar-H), 7.92 (ddd, ^3J(H,H) = 8.16 Hz, ^4J(H,H) = 2.17 Hz, ^4J(H,H) = 2.20 Hz, 1H; Ar-H), 7.43 (t, ^3J(H,H) = 8.20 Hz, 1H; Ar-H), 7.25-7.21 (m, 4H; Ar-H), 7.21-7.19 (m, 1H; Ar-H), 5.81 (s, 1H), 3.00 (d, ^3J(H,H) = 5.25 Hz, 3H; CH_3), 1.86 ppm (s, 3H; CH_3). **^{13}C NMR** (150 MHz, $CDCl_3$) δ 175.21, 165.68, 164.65, 148.24, 137.98, 137.81, 129.68, 129.08, 128.73, 127.90, 120.20, 117.18, 107.14, 60.97, 30.17, 15.21 ppm. **HRMS** (ESI-TOF MS/MS) *m/z* $[M + H]^+$ calcd for $C_{19}H_{17}N_3O_4$: 352.1297; found: 352.1280.

2. Determination of crystal structure of 5a

X-ray intensity data for **5a** were collected at 293(2) K on an Agilent SuperNova diffractometer, equipped with an Eos CCD detector, using MoKα radiation (λ = 0.71073 Å). The images were processed (unit cell determination, intensity data integration, correction for Lorentz and polarization effects, and empirical absorption correction) using CrysAlisPRO [4]. Using Olex2 [5], the structure was solved with the ShelXT [6] structure solution program using Intrinsic Phasing and refined with the ShelXL [7] refinement package using full-matrix least-squares minimization on F^2. Hydrogen atom H15 was located in difference density maps and subsequently refined freely. All other hydrogen atoms were placed in idealized positions and refined in the 'riding mode'. Non-hydrogen atoms were refined anisotropically and hydrogen atoms with isotropic temperature factors were fixed at 1.2 times U_{eq} of the parent atoms (1.5 for methyl and OH groups). A summary of the crystal data and structure refinement is given in Table S1. Crystallographic data for the structure reported herein have been deposited with the Cambridge Crystallographic Data Centre as supplementary publication numbers CCDC 2378509. The data can be obtained free of charge via http://www.ccdc.cam.ac.uk/conts/retrieving.html, or from the Cambridge Crystallographic Data Center, 12 Union Road, Cambridge CB2 1EZ, UK; fax: +441223 336 033; or e-mail: deposit@ccdc.cam.ac.uk.

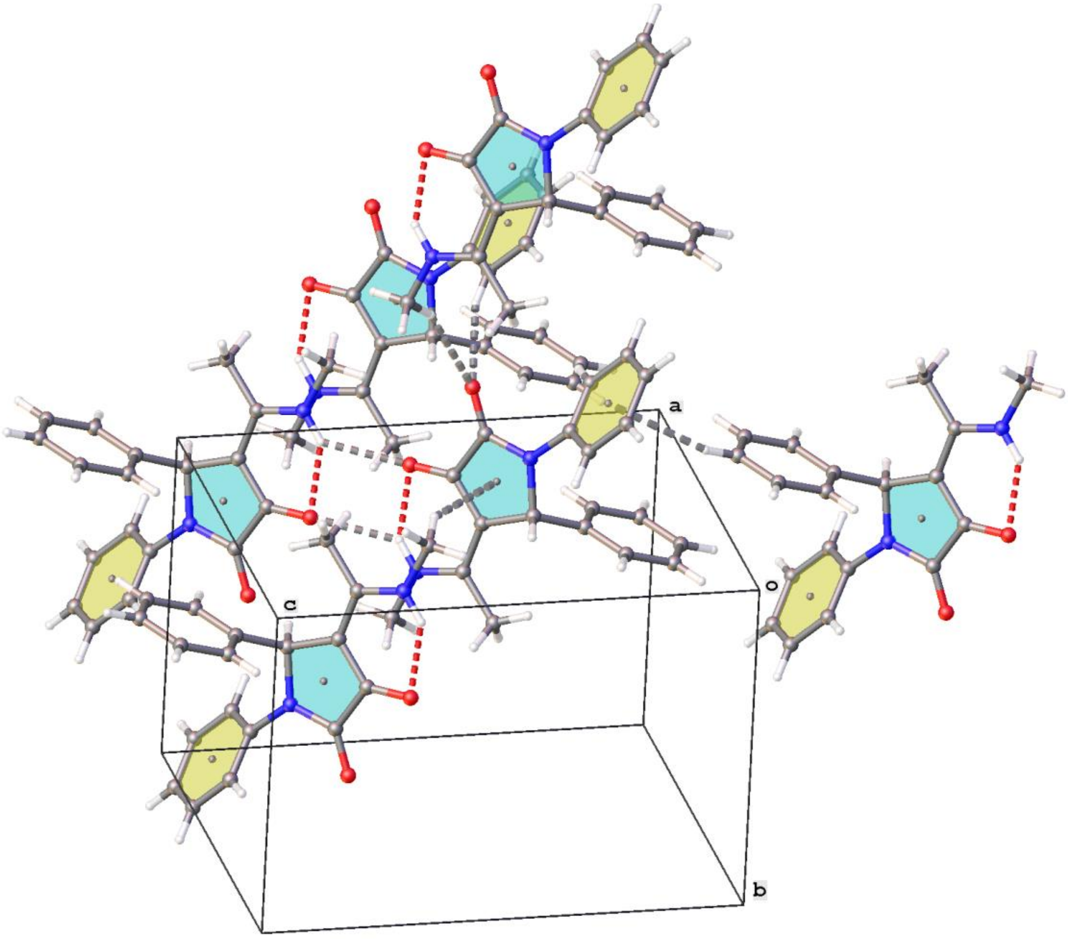

Figure S1. Partial packing diagram for **5a**. Intramolecular hydrogen bonds are shown as red dashed lines, intermolecular C-H···O and C–H···π interactions are shown as grey dashed lines

Table S1. Crystal data and structure refinement for **5a**

Empirical formula	$C_{19}H_{18}N_2O_2$
Formula weight	306.35
Temperature/K	293(2)
Crystal system	triclinic
Space group	P-1
a/Å	6.2252(2)
b/Å	9.1920(4)
c/Å	15.5092(6)
α/°	104.409(3)
β/°	93.958(3)
γ/°	108.567(4)
Volume/Å^3	804.12(6)
Z	2
ρ_{calc}g/cm^3	1.265
μ/mm^{-1}	0.083
F(000)	324.0
Crystal size/mm^3	0.5 × 0.25 × 0.05
Radiation	MoKα (λ = 0.71073 Å)
2Θ range for data collection/°	4.866 to 52.734
Index ranges	$-7 \le h \le 7$, $-11 \le k \le 11$, $-19 \le l \le 19$
Reflections collected	16495
Independent reflections	3285 [R_{int} = 0.0221, R_{sigma} = 0.0177]
Data/restraints/parameters	3285/0/214
Goodness-of-fit on F^2	1.101
Final R indexes [I>=2σ (I)]	R_1 = 0.0461, wR_2 = 0.1154
Final R indexes [all data]	R_1 = 0.0568, wR_2 = 0.1222
Largest diff. peak/hole / e Å^{-3}	0.22/-0.17

Table S2. Hydrogen bond geometry (Å, °) for **5a**

	D-H	H···A	D···A	D–H···A
N15-H15…O13	0.90(3)	2.01(3)	2.739(2)	138(2)
N15-H15…O13^{i}	0.90(3)	2.31(2)	3.000(2)	134(2)
C7-H7…O12^{ii}	0.93	2.29	3.185(3)	161
C16-H16A…O12^{iii}	0.96	2.47	3.353(3)	152
C16-H16C…*Cg*1^{iv}	0.96	2.85	3.677(2)	145
C21-H21…*Cg*2^{v}	0.93	2.85	3.749(2)	163

*Cg*1 and *Cg*2 are the centroids of rings N1/C2-C5 (blue in Figure S1) and C6-C11 (yellow in Figure S1), respectively. Symmetry codes: (i) 1-x, 1-y, 1-z ; (ii) -1+x, y, z ; (iii) x, 1+y, z ; (iv) -x, 1-y, 1-z ; (v) -1-x, -y, -z.

3. Bioassay for NO inhibition

3.1. Materials

Lipopolysaccharides (LPS) from *Escherichia coli*, sodium nitrite, sulfanilamide, *N*-1-napthylethylenediamine dihydrochloride, 3-(4,5-dimethylthiazol-2-yl)-2,5-diphenyl-tetrazolium bromide (MTT), 4-(2-hydroxyethyl)-1-piperazineethanesulfonic acid (HEPES), dimethyl sulfoxide (DMSO), L-glutamine, dexamethasone and sodium pyruvate were purchased from Sigma Aldrich, Promega, Gibco, Invitrogen. Fetal bovine serum (FBS) and Dulbecco's Modified Eagle Medium (DMEM) were provided by Life Technologies, Inc., (Gaithersburg, MD, USA). RAW 264.7 cell lines were provided by Prof. Dr Domenico Delfino, University of Perugia, Perugia, Italy.

3.2. Cell culture

RAW 264.7 cells were cultured in DMEM which supplemented with HEPES (10 mM), sodium pyruvate (1 mM), L-glutamine (2mM) and fetal bovine serum (FBS 10%). Cells were subcultured every three days with the ratio of 1:3 and maintained in the incubator at 37 °C and 5% CO_2 [8,9].

3.3. NO inhibition assay

The inhibition capability of five pyrrolidine-2,3-diones **5a–e** on NO production was evaluated on LPS-stimulated RAW 264.7 cells. RAW 264.7 cells were seeded in 96-well culture plates at the concentration of 2×10^6 cells/mL and incubated in a chamber at 37 °C and 5% CO_2 for 24 hours. Then, the culture medium was replaced by DMEM without FBS. To each well of 96-well culture plates, samples at different concentrations (0.8, 4.0, 20, and 100 μM) were added and subsequently, incubated for 2 hours under identical conditions. Then, RAW 264.7 cells were activated by lipopolysaccharides (LPS, 1.0 μg/mL) for 24 hours to produce nitric oxide (NO). A dexamethasone solution at different concentrations (0.8, 4.0, 20, and 100 μM) was used as the positive control and DMSO solution (10%) as the negative control. Nitrite (NO_2^-) concentration, considered as the indicator of NO production, was determined via Griess Reagent System (Promega Cooperation). Briefly, cell culture medium (100 μL) and Griess reagent (100 μL) were mixed in a 96-well plate and incubated in a chamber at room temperature for 10 minutes. Then, nitrite concentration was measured at the wavelength of 540 nm in a microplate reader (BioTek Elx800). DMEM without FBS was used as the blank well. Standard curve of sodium nitrite ($NaNO_2$) was used to determine

the concentration of nitrite in all tested samples as compared with negative control sample (LPS). The inhibition capability of nitric oxide (NO) production of all tested samples was calculated by the following formula [10]:

Inhibition (%) = 100 – [concentration of NO in tested samples/concentration of NO in negative control sample]*100.

All experiments were repeated three times and the half-maximum inhibitory concentrations (IC_{50}) were calculated via the Table Curve Version 4.0 programme (Systat Software Inc., San Jose, CA, USA).

3.4. MTT cell viability assay

After removal of the supernatant for the determination of nitric oxide content, 90 μL of cell culture medium and 10 μL of MTT solution (5 mg/mL) were added to each well of 96-well culture plate. The plate was incubated at 37 °C and 5% CO_2 for 4 hours and then, cell culture medium was removed from all wells of 96-well plate. Formazan crystals in each well were dissolved by 50 μL of DMSO 100% and optical density (OD) was recorded at the wavelength of 540 nm by a microplate reader (BioTek Elx800). Survival rate of RAW 264.7 cells was determined by the following formula [11-13]:

$$\text{Cell viability (\%)} = \frac{OD(sample) - OD(blank)}{OD(DMSO) - OD(blank)}\ x\ 100$$

Table S3. Inhibitory capability of pyrrolidine-2,3-diones **5a–e** on NO production

**Concentration (μM)**	**5a**				**5b**			
	**NO inhibition (%)**		**Cell viability (%)**		**NO inhibition (%)**		**Cell viability (%)**	
	**Mean**	**SD**	**Mean**	**SD**	**Mean**	**SD**	**Mean**	**SD**
**100**	53.65	1.56	90.46	3.40	50.22	1.81	91.11	1.73
**20**	32.08	1.21	101.38	1.72	28.88	1.53	100.03	2.27
**4**	9.31	0.84			7.24	0.42		
**0.8**	2.04	0.21			1.11	0.18		
**IC_{50}**	**78.65±6.88**		-		**95.66±9.93**		-	
	**5e**				**5d**			

Concentration (µM)	NO inhibition (%)		Cell viability (%)		NO inhibition (%)		Cell viability (%)	
	Mean	SD	Mean	SD	Mean	SD	Mean	SD
**100**	63.65	2.02	90.35	1.58	38.41	1.80	95.74	1.09
**20**	39.22	1.84	93.57	2.84	23.79	1.68	98.45	1.75
**4**	18.82	0.85			8.37	0.83		
**0.8**	5.25	0.47			1.88	0.14		
**IC_{50}**	**43.69±5.26**		-		**>100**		-	

Concentration (µM)	5c				Dexamethasone			
	NO inhibition (%)		Cell viability (%)		NO inhibition (%)		Cell viability (%)	
	Mean	SD	Mean	SD	Mean	SD	Mean	SD
**100**	23.76	1.87	90.75	2.31	85.04	2.02	94.82	2.91
**20**	15.63	1.31	92.49	2.17	53.31	1.58	101.58	1.54
**4**	10.00	0.84			42.73	1.13		
**0.8**	3.25	0.46			32.31	0.72		
**IC_{50}**	**>100**		-		**13.25±1.39**		-	

Table S4. Drug-likeness properties of studied compounds

Rules	Studied compounds				
	**5a**	**5b**	**5c**	**5d**	**5e**
Lipinski	Yes	Yes	Yes	Yes	Yes
Ghose	Yes	Yes	Yes	Yes	Yes
Veber	Yes	Yes	Yes	Yes	Yes
Egan	Yes	Yes	Yes	Yes	Yes
Muegge	Yes	Yes	Yes	Yes	Yes

4. ADMET properties

The ADMET properties were predicted using the pkCSM online tool (http://biosig.unimelb.edu.au/pkcsm/), which utilizes graph-based signatures to estimate pharmacokinetic and toxicity parameters. The output data from pkCSM, including values for absorption, distribution, metabolism, excretion, and toxicity (ADMET), are provided in Table S5 For more details, the interested reader is referred to the official pkCSM theory page: https://biosig.lab.uq.edu.au/pkcsm/theory.

Table S5: Data from pkCSM, including values for absorption, distribution, metabolism, excretion, and toxicity (ADMET) of the studied compounds.

Property	Model Name	Unit	5a	5b	5c	5d	5e
			Predicted Value				
**Absorption**	Water solubility	Numeric (log mol/L)	-3.706	-3.996	-4.475	-4.43	-4.577
	Caco2 permeability	Numeric (log Papp in 10^{-6} cm/s)	1.36	1.358	1.472	1.082	1.475
	Intestinal absorption (human)	Numeric (% Absorbed)	96.709	96.684	90.093	95.159	90.294
	Skin Permeability	Numeric (log Kp)	-2.68	-2.676	-2.725	-2.7	-2.722
	P-glycoprotein substrate	Categorical (Yes/No)	No	No	No	No	No
	P-glycoprotein I inhibitor	Categorical (Yes/No)	Yes	Yes	Yes	Yes	Yes
	P-glycoprotein II inhibitor	Categorical (Yes/No)	No	No	No	No	No
**Distribution**	VDss (human)	Numeric (log L/kg)	-0.088	0.162	-0.185	0.139	-0.183
	Fraction unbound (human)	Numeric (Fu)	0	0	0	0	0
	BBB permeability	Numeric (log BB)	0.22	0.204	-0.62	0.189	-0.622
	CNS permeability	Numeric (log PS)	-2.143	-2.07	-2.343	-2.007	-2.356
**Metabolism**	CYP2D6 substrate	Categorical (Yes/No)	No	No	No	No	No
	CYP3A4 substrate	Categorical (Yes/No)	Yes	Yes	Yes	Yes	Yes
	CYP1A2 inhibitior	Categorical (Yes/No)	No	No	Yes	No	Yes
	CYP2C19 inhibitior	Categorical (Yes/No)	No	Yes	Yes	Yes	Yes
	CYP2C9 inhibitior	Categorical (Yes/No)	No	Yes	Yes	Yes	Yes
	CYP2D6 inhibitior	Categorical (Yes/No)	No	No	No	No	No
	CYP3A4 inhibitior	Categorical (Yes/No)	No	No	No	No	No
**Excretion**	Total Clearance	Numeric (log ml/min/kg)	0.304	0.306	0.322	-0.077	0.274
	Renal OCT2 substrate	Categorical (Yes/No)	No	No	No	No	No
**Toxicity**	AMES toxicity	Categorical (Yes/No)	No	No	Yes	No	Yes
	Max. tolerated dose (human)	Numeric (log mg/kg/day)	0.644	0.648	0.032	0.629	-0.044
	hERG I inhibitor	Categorical (Yes/No)	No	No	No	No	No
	hERG II inhibitor	Categorical (Yes/No)	No	No	No	No	No
	Oral Rat Acute Toxicity (LD50)	Numeric (mol/kg)	2.281	2.205	2.935	2.395	2.9
	Oral Rat Chronic Toxicity (LOAEL)	Numeric (log mg/kg_bw/day)	1.653	1.648	1.783	1.763	1.892

	Hepatotoxicity	Categorical (Yes/No)	Yes	Yes	Yes	Yes	Yes
	Skin Sensitization	Categorical (Yes/No)	No	No	No	No	No
	T. Pyriformis toxicity	Numeric (log ug/L)	0.773	0.849	0.389	0.831	0.409
	Minnow toxicity	Numeric (log mM)	1.721	1.175	0.984	0.811	0.971

References

[1] Nguyen, N. T.; Dai, V. V.; Tri, N. N.; Van Meervelt, L.; Trung, N. T.; Dehaen, W. *Beilstein J. Org. Chem.* **2022,** 18, 1140-1153. doi: 10.3762/bjoc.18.118

[2] Nguyen, N. T.; Dai, V. V. *Vietnam Journal of Chemistry and Applications* **2022,** 1B, 131-136.

[3] Nguyen, N. T.; Dai, V. V. *The University of Danang - Journal of Science and Technology* **2023,** 61-66. doi: 10.31130/ud-jst.2023.519E

[4] P. CrysAlis, Oxfordshire, UK, 2012

[5] Dolomanov, O.; Bourhis, L.; Gildea, R.; Howard, J.; Puschmann H. *J. Appl. Crystallogr*. **2009,** 42, 339-341.

[6] Sheldrick, G. M. *Acta Crystallogr. A: Found. Adv*. **2015, 71**, 3-8.

[7] Sheldrick, G. M. *Acta crystallogr., C Struct. chem*. **2015,** 71, 3-8.

[8] Tsai, P.-J.; Tsai, T.-H.; Yu, C.-H.; Ho, S.-C. *Food Chem*. **2007,** 103, 181-187

[9] Joo, T.; Sowndhararajan, K.; Hong, S.; Lee, J.; Park, S.-Y.; Kim, S.; Jhoo, J.-W. *Saudi J. Biol. Sci*. **2014,** 21, 427-435.

[10] Cheenpracha, S.; Park, E.-J.; Rostama, B.; Pezzuto, J. M.; Chang, L. C. *Mar. Drugs* **2010,** 8, 429-437.

[11] Bhavikatti, S. K.; Karobari, M. I.; Zainuddin, S. L. A.; Marya, A.; Nadaf, S. J.; Sawant, V. J.; Patil, S. B.; Venugopal, A.; Messina, P.; Scardina, G. A. *Int. J. Environ. Res. Public Health*. **2021,** 18, 7162

[12] Diep, T. T.; Van Dung, L.; Van Trung, P.; Hoai, N. T.; Thao, D. T.; Uyen, N. T. T.; Linh, T. T. H.; Truc, H. T.; Tran, L. T. T. *J. Essent. Oil-Bear. Plants* **2023,** 26, 1460-1472.

[13] Trinh Thi, D.; Luong Van, D.; Phung Van, T.; Nguyen Thi, H.; Do Thi, T.; Nguyen Thi To, U.; Tran Thi Hoai, L.; Dang Vinh, K.; Huynh, T. T.; Le Thi Thanh, T. *Nat. Prod. Res*. **2024,** 38, 1834-1843.

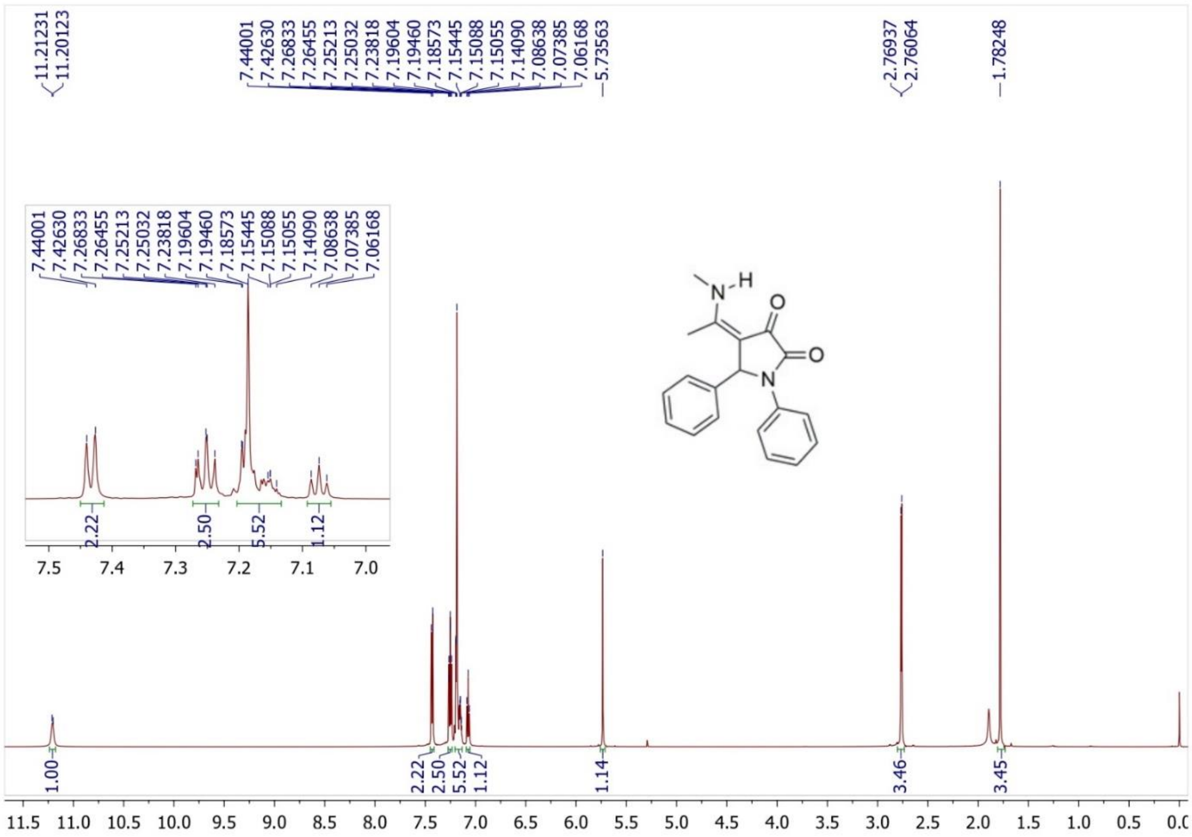

Figure S2. ^{1}H NMR spectrum of 4-(1-methylamino)ethylidene-1,5-diphenyl pyrrolidine-2,3-dione (**5a**)

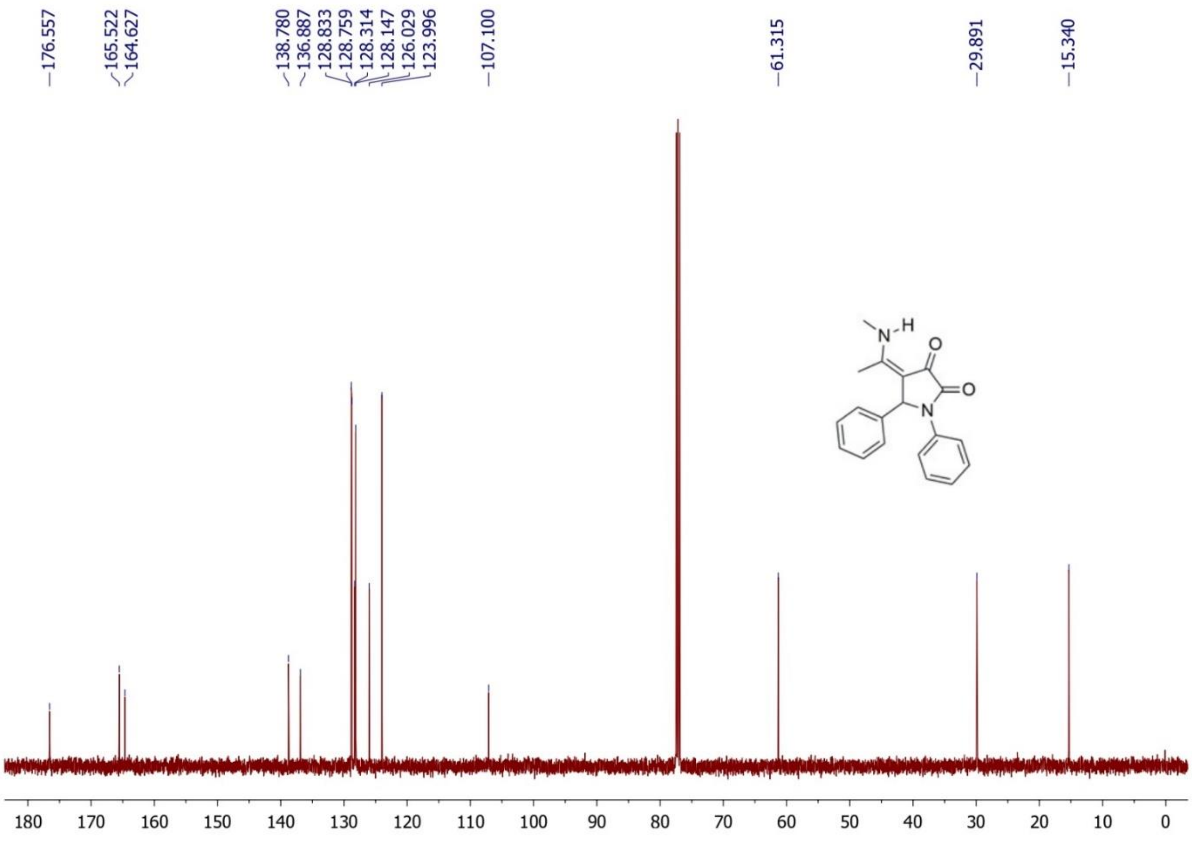

Figure S3. ^{13}C NMR spectrum of 4-(1-methylamino)ethylidene-1,5-diphenyl pyrrolidine-2,3-dione (**5a**)

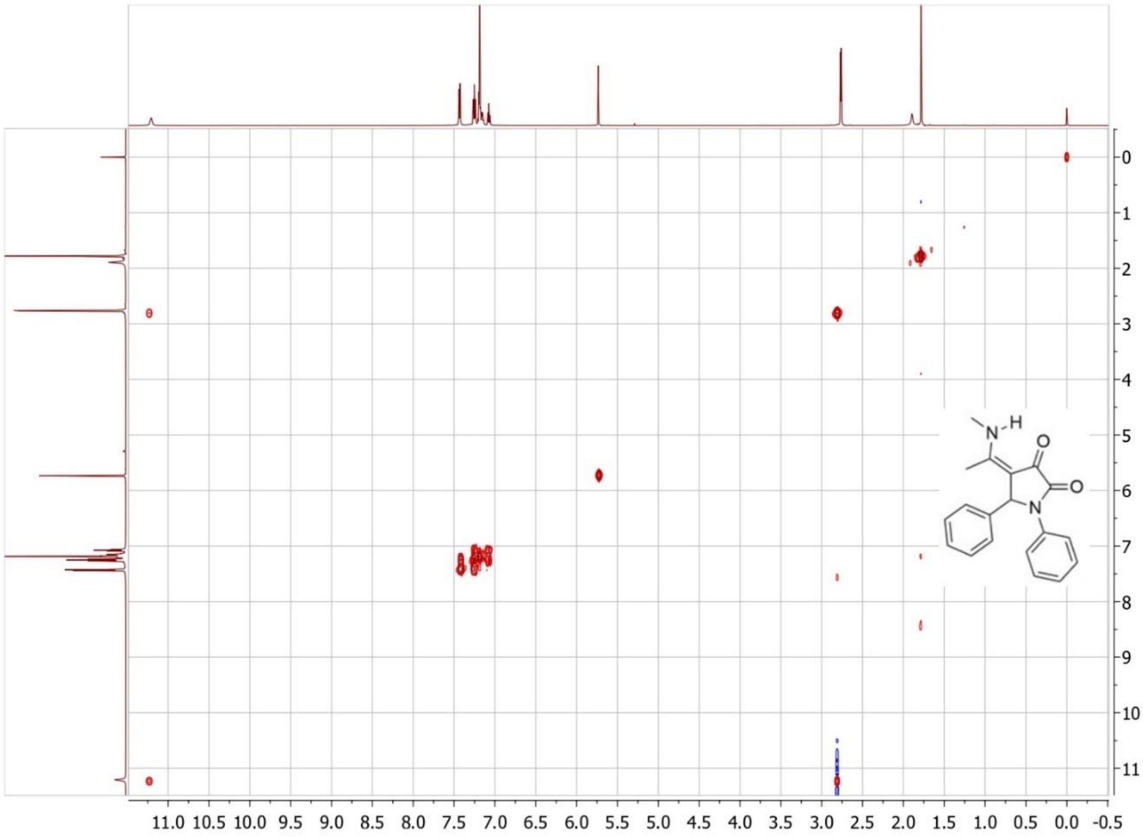

Figure S4. 2D ^{1}H-^{13}C HSQC spectrum of 4-(1-methylamino)ethylidene-1,5-diphenyl pyrrolidine-2,3-dione (**5a**)

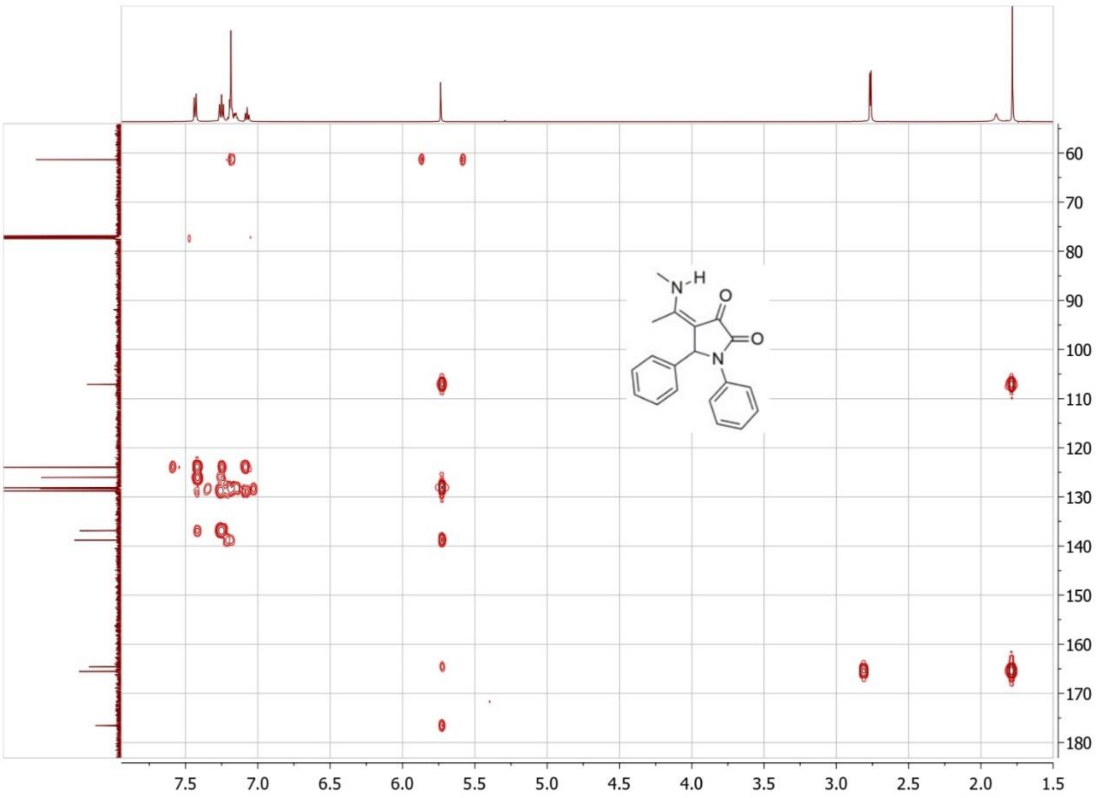

Figure S5. 2D ^{1}H-^{13}C HMBC spectrum of 4-(1-methylamino)ethylidene-1,5-diphenylpyrrolidine-2,3-dione (**5a**)

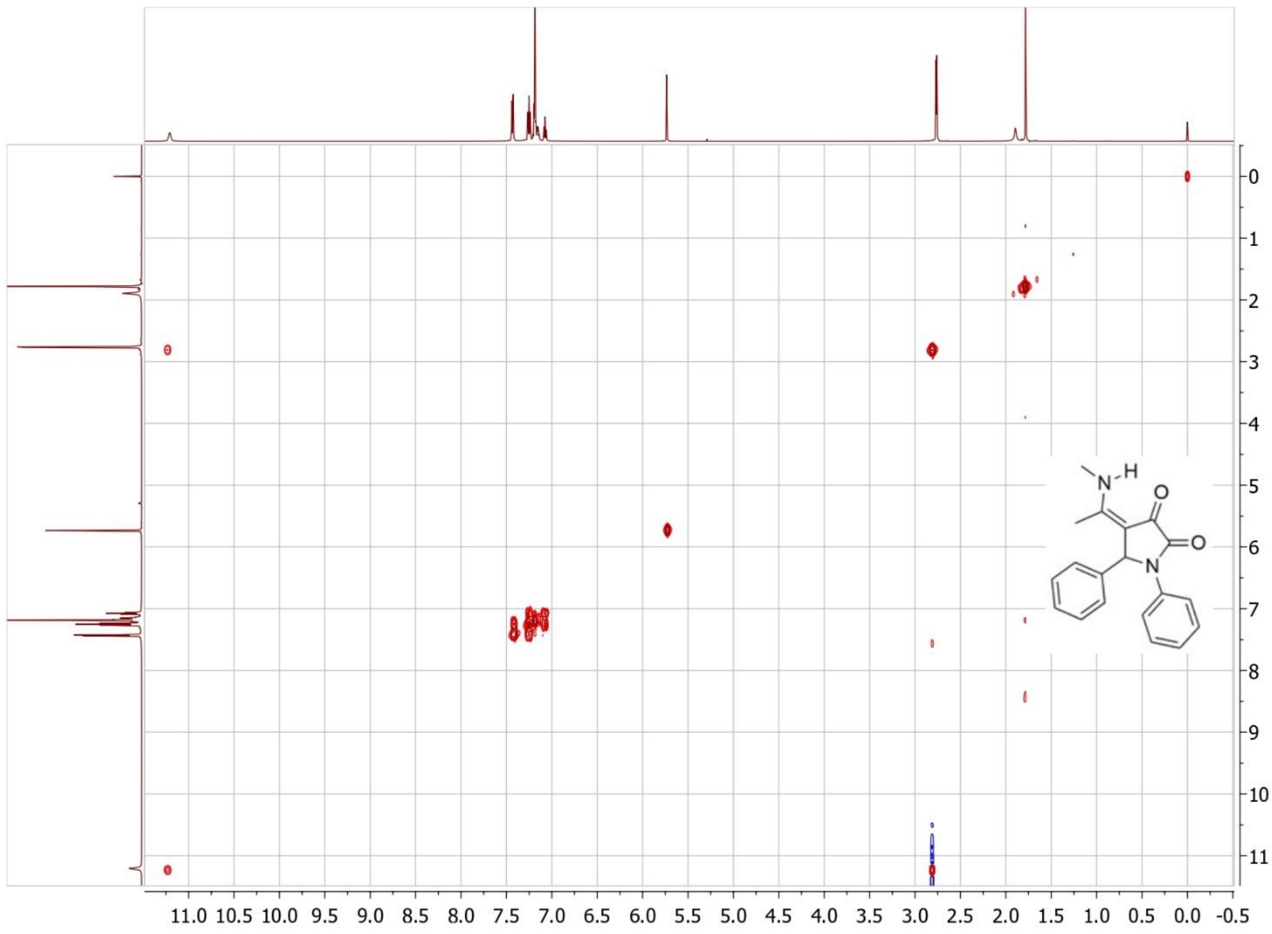

Figure S6. 2D ^1H–^1H COSY spectrum of 4-(1-methylamino)ethylidene-1,5-diphenylpyrrolidine-2,3-dione (**5a**)

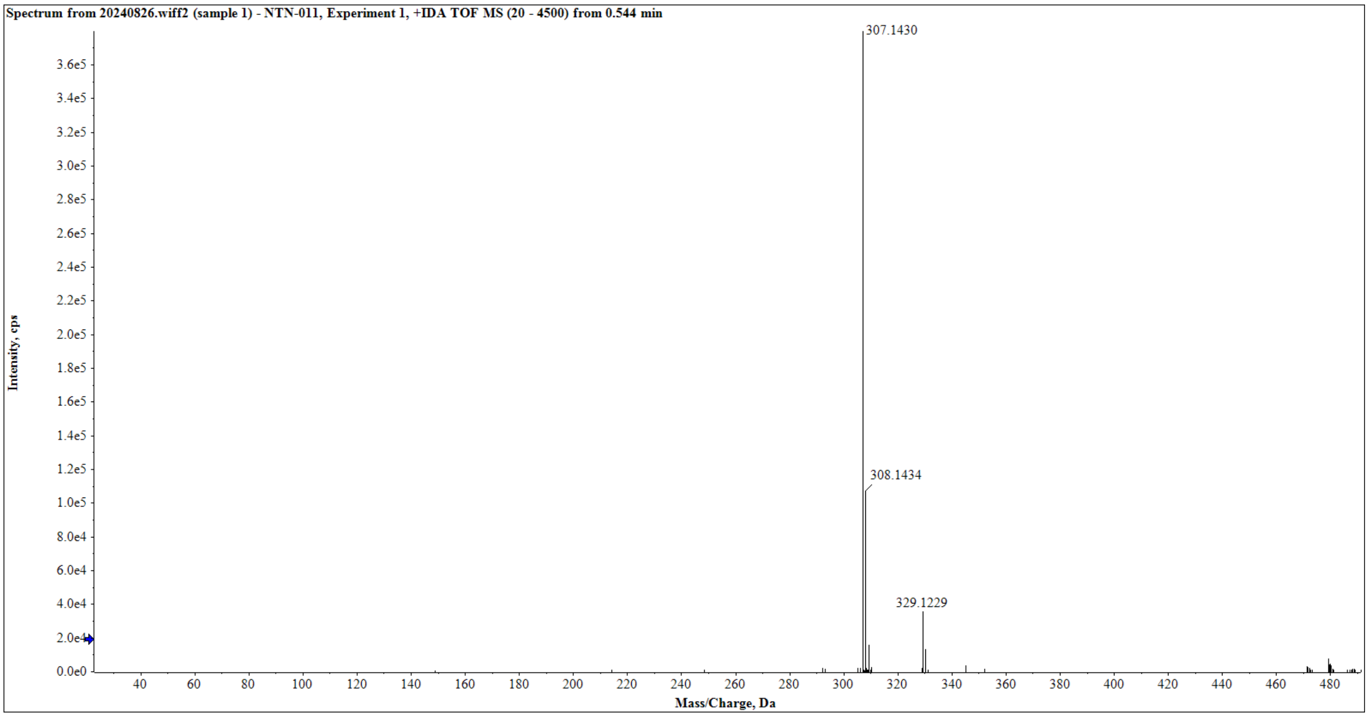

Figure S7. ESI-HRMS spectrum of 4-(1-methylamino)ethylidene-1,5-diphenylpyrrolidine-2,3-dione (**5a**)

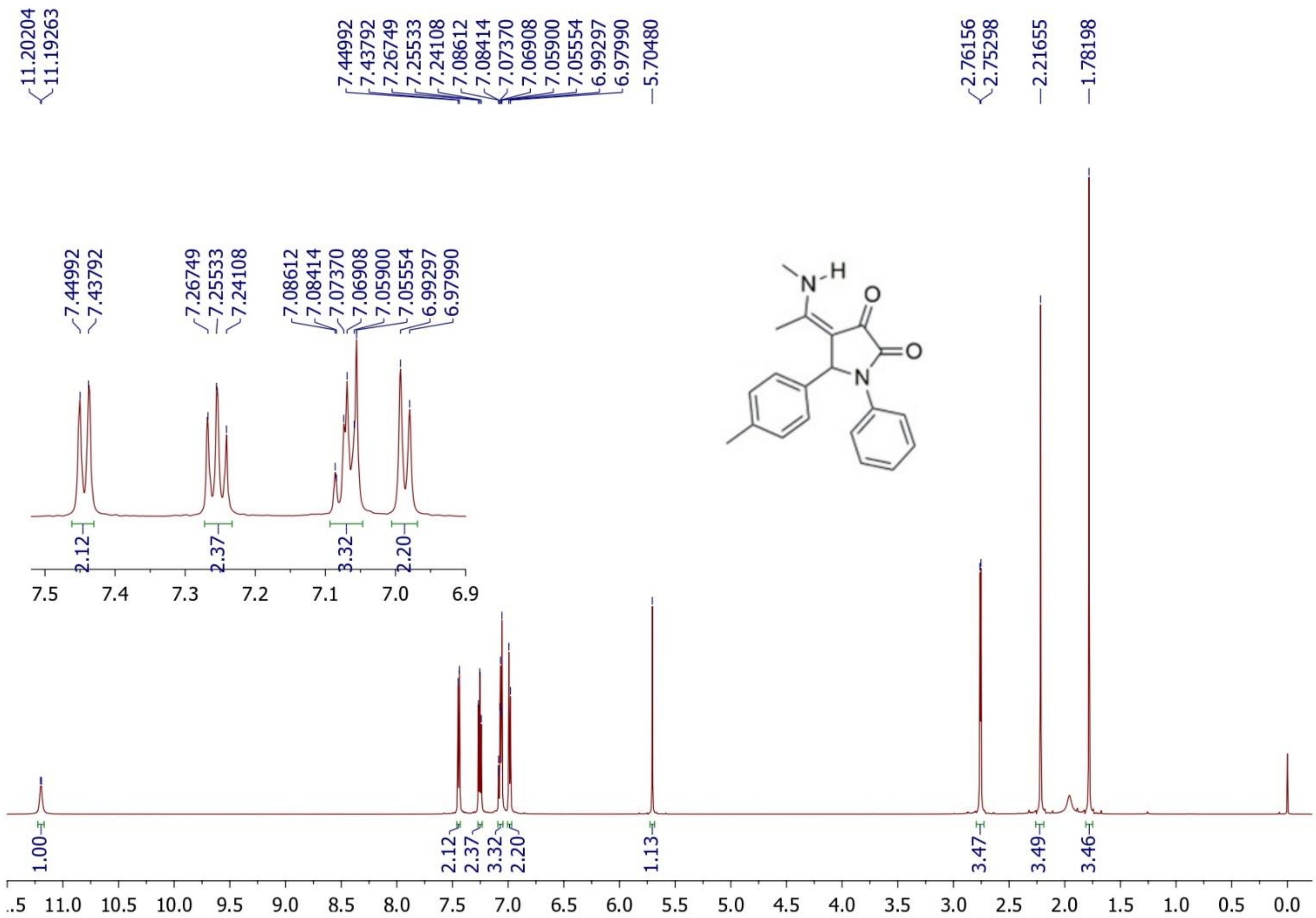

Figure S8. ^{1}H NMR spectrum of 5-(4-methylphenyl)-4-(1-methylamino)ethylidene-1-phenylpyrrolidine-2,3-dione (**5b**)

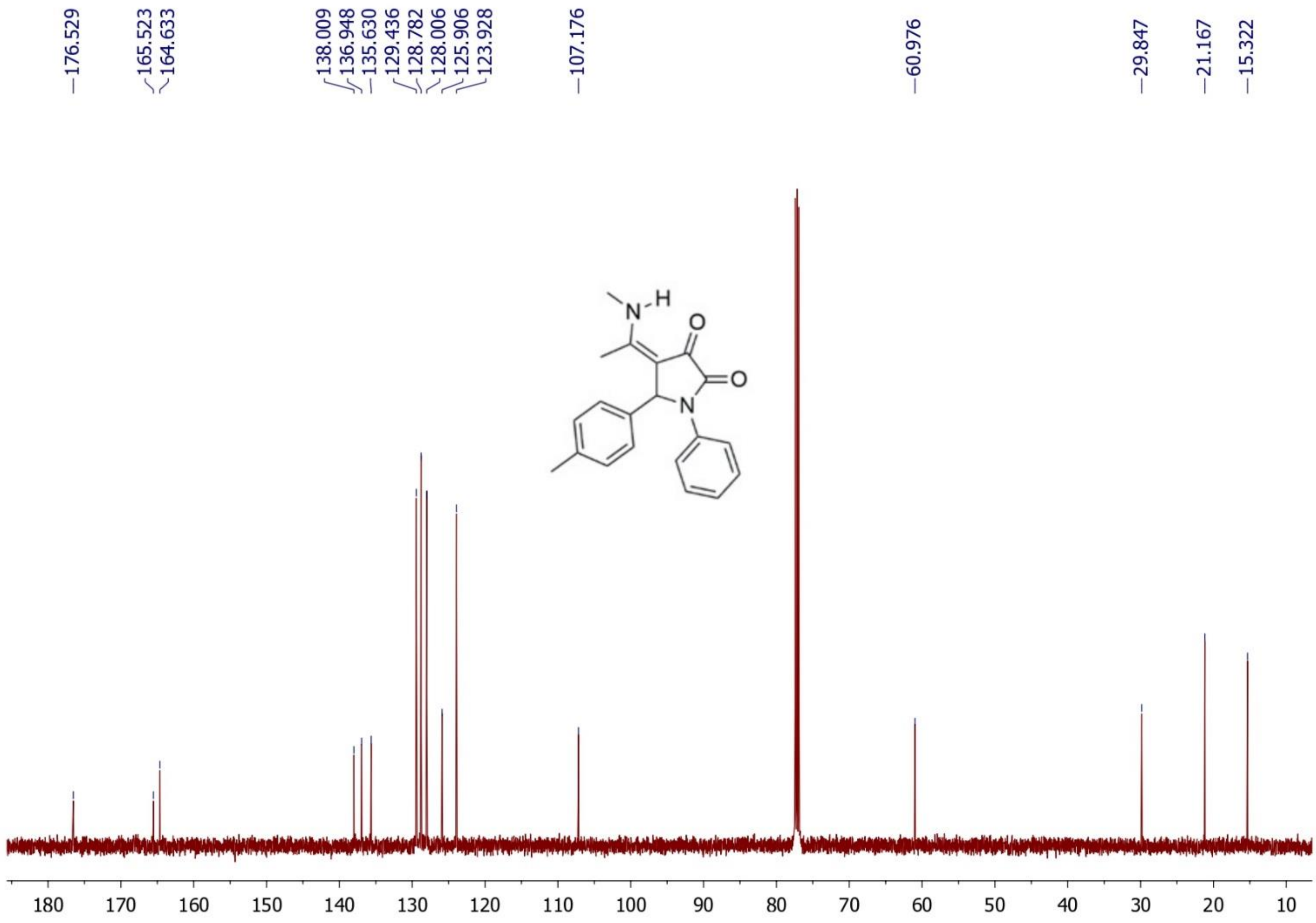

Figure S9. ^{13}C NMR spectrum of 5-(4-methylphenyl)-4-(1-methylamino)ethylidene-1-phenylpyrrolidine-2,3-dione (**5b**)

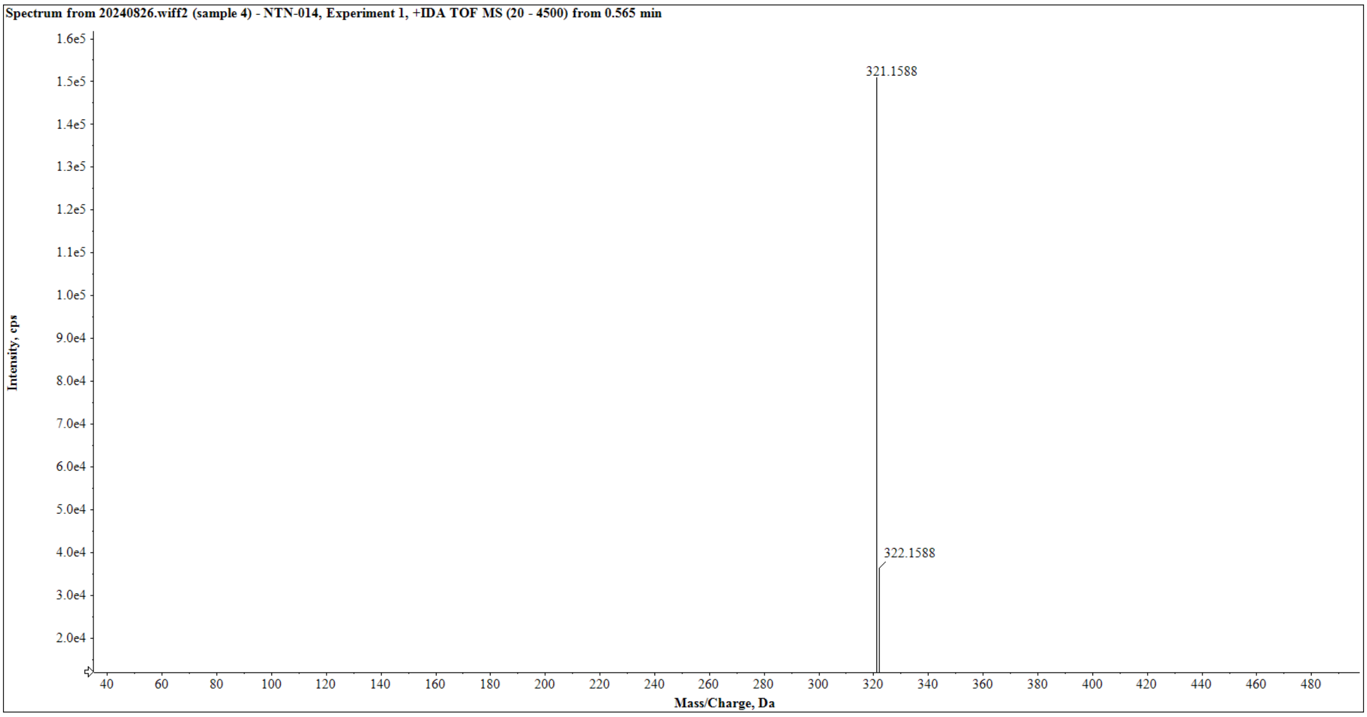

Figure S10. ESI-HRMS spectrum of 5-(4-methylphenyl)-4-(1-methylamino)ethylidene-1-phenylpyrrolidine-2,3-dione (**5b**)

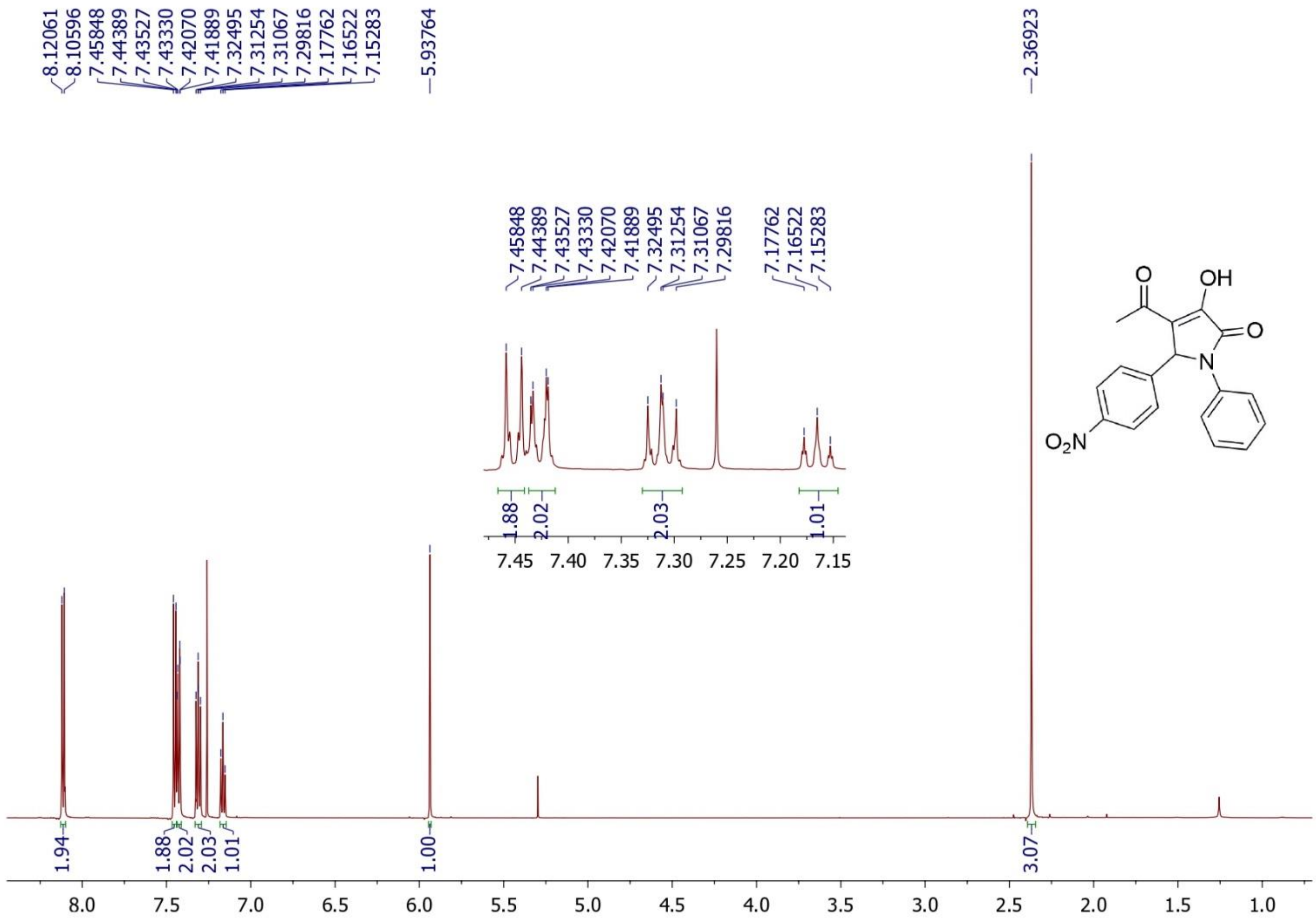

Figure S11. ^{1}H NMR spectrum of 4-acetyl-3-hydroxy-1-phenyl-5-(4-nitrophenyl)-3-pyrroline-2-one (**1c**)

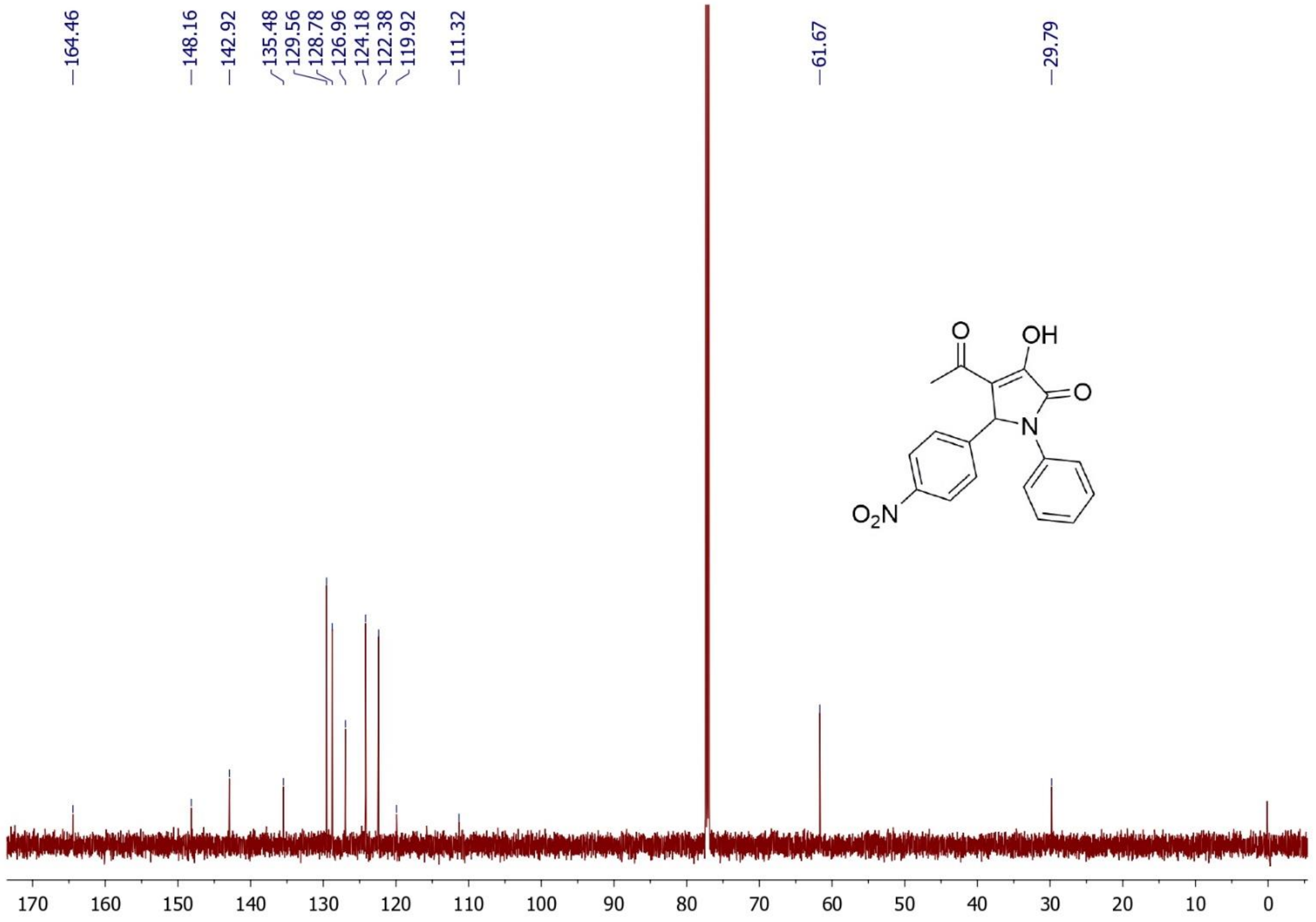

Figure S12. ^{13}C NMR spectrum of 4-acetyl-3-hydroxy-1-phenyl-5-(4-nitrophenyl)-3-pyrroline-2-one (**1c**)

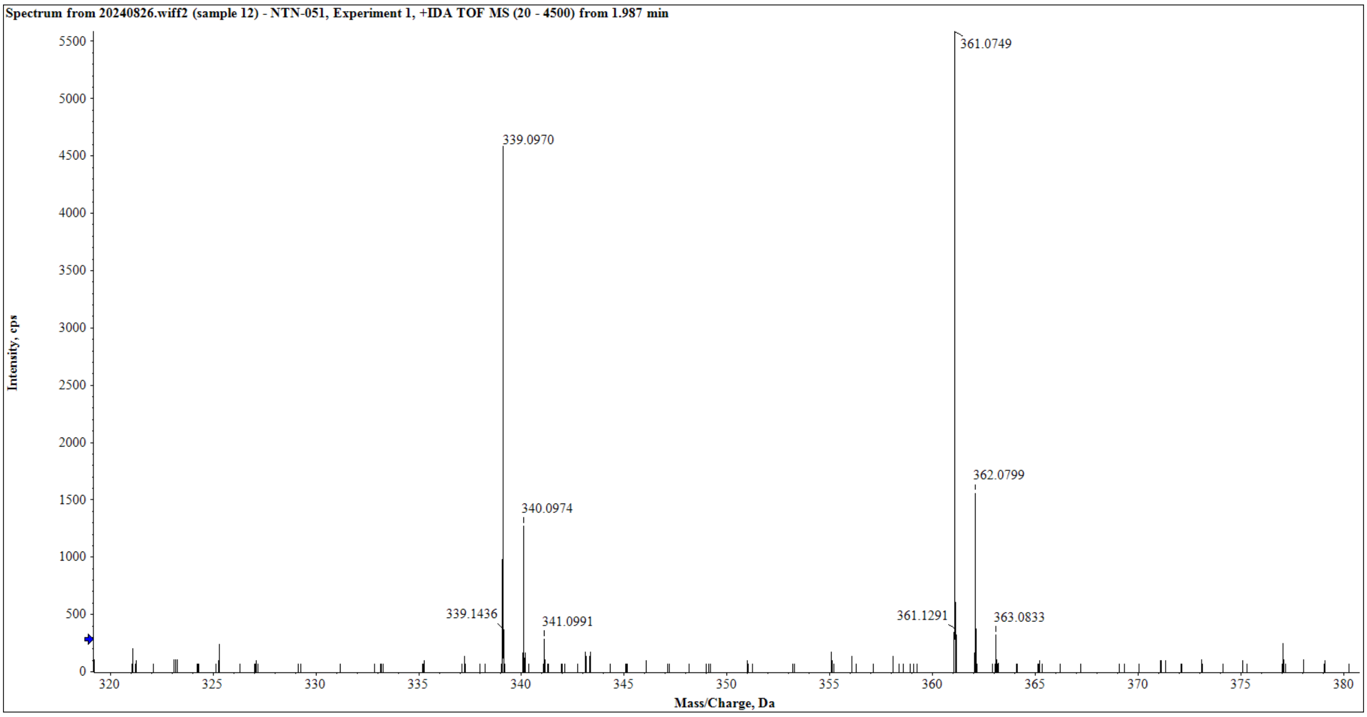

Figure S13. ESI-HRMS spectrum of 4-acetyl-3-hydroxy-1-phenyl-5-(4-nitrophenyl)-3-pyrroline-2-one (**1c**)

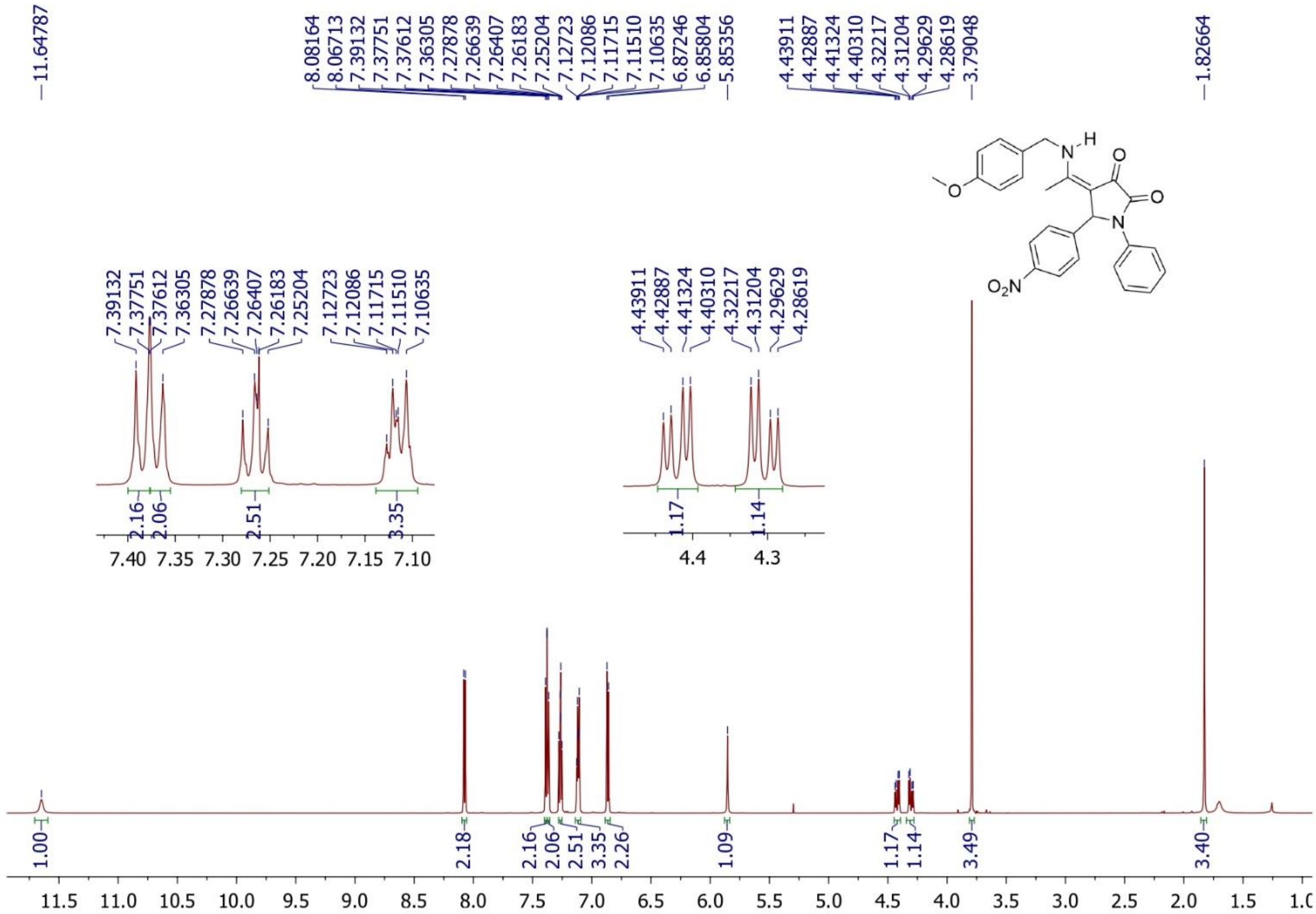

Figure S14. ^{1}H NMR spectrum of 4-[1-(4-methoxybenzyl)amino]ethylidene-1-phenyl-5-(4-nitrophenyl)pyrrolidine-2,3-dione (**3c**)

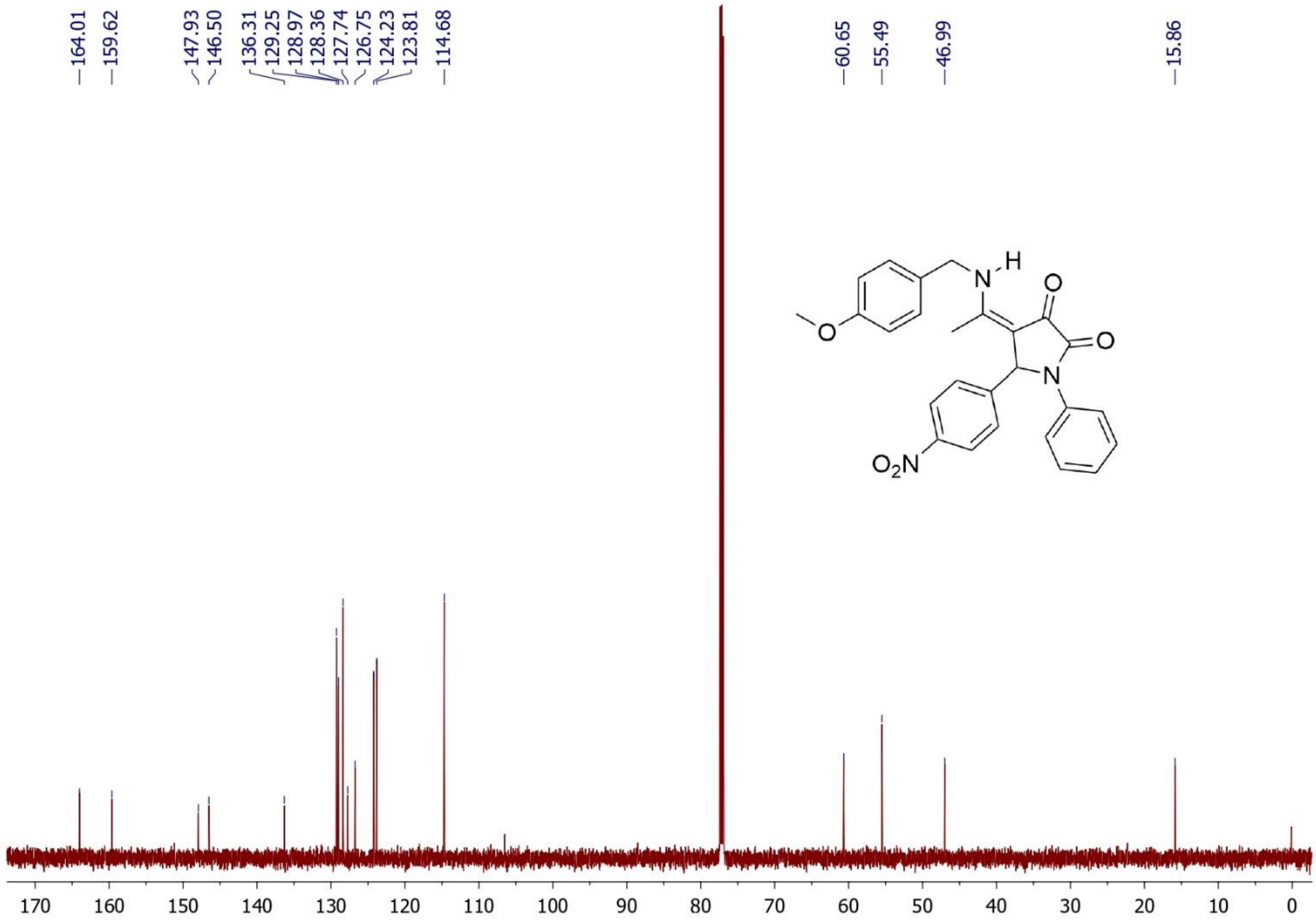

Figure S15. ^{13}C NMR spectrum of 4-[1-(4-methoxybenzyl)amino]ethylidene-1-phenyl-5-(4-nitrophenyl)pyrrolidine-2,3-dione (**3c**)

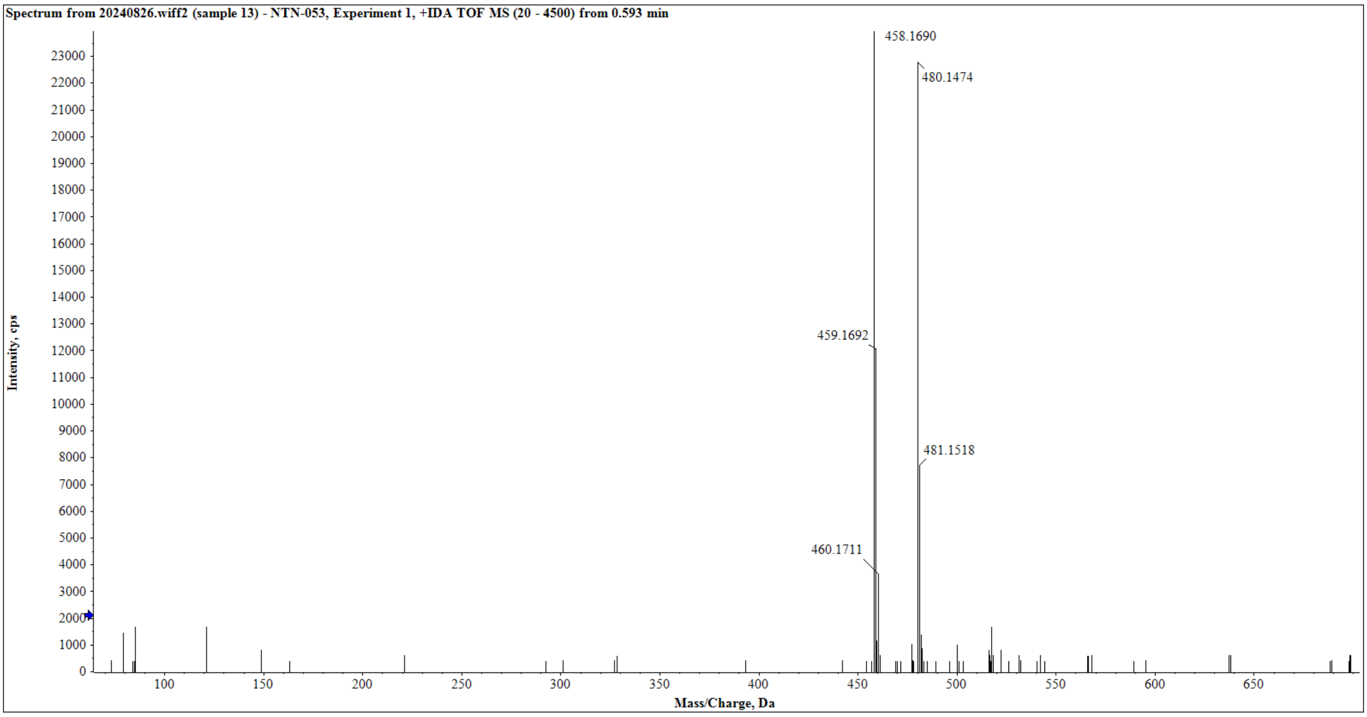

Figure S16. ESI-HRMS spectrum of 4-[1-(4-methoxybenzyl)amino]ethylidene-1-phenyl-5-(4-nitrophenyl)pyrrolidine-2,3-dione (**3c**)

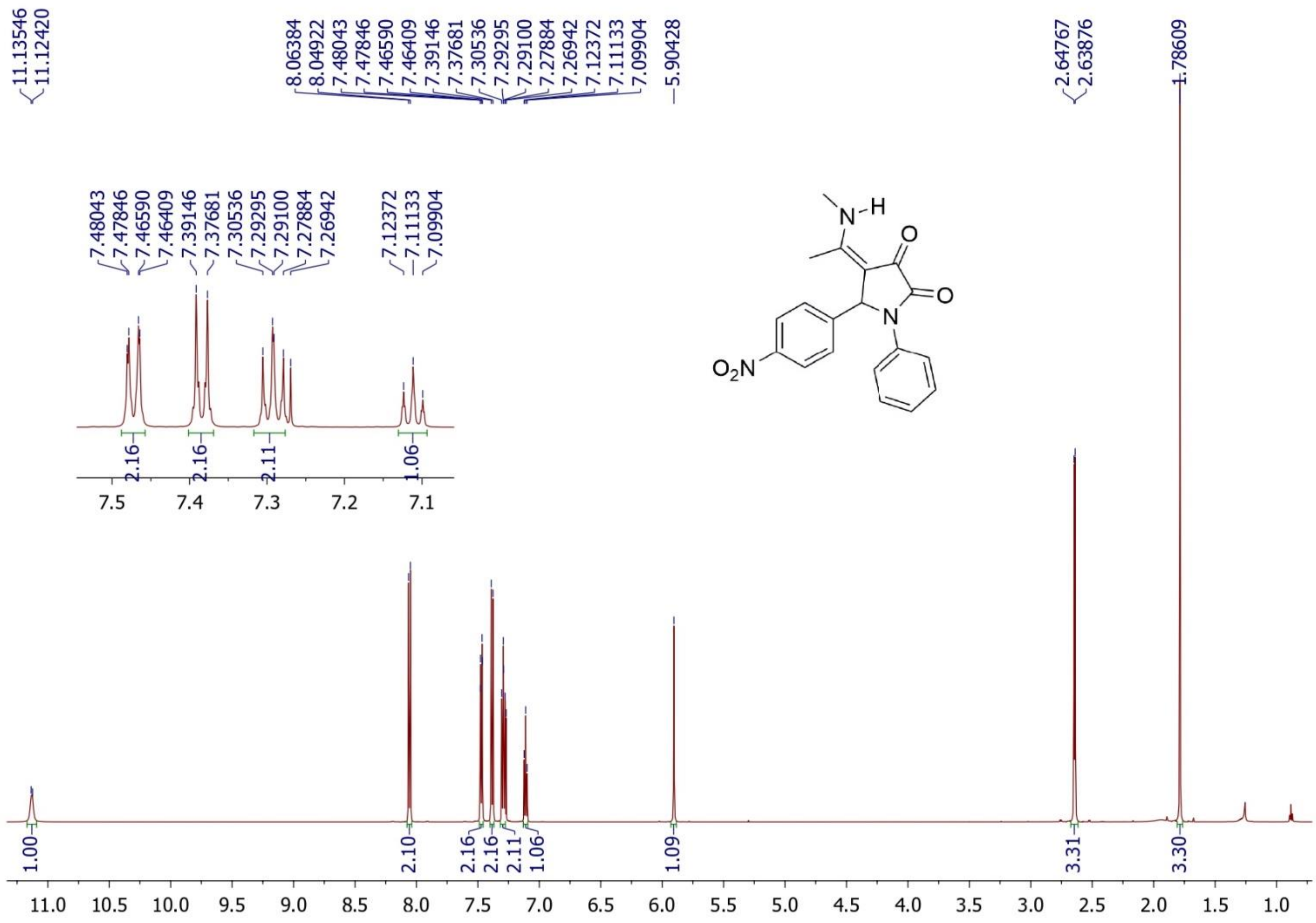

Figure S17. ^{1}H NMR spectrum of 4-(1-methylamino)ethylidene-1-phenyl-5-(4-nitrophenyl)pyrrolidine-2,3-dione (**5c**)

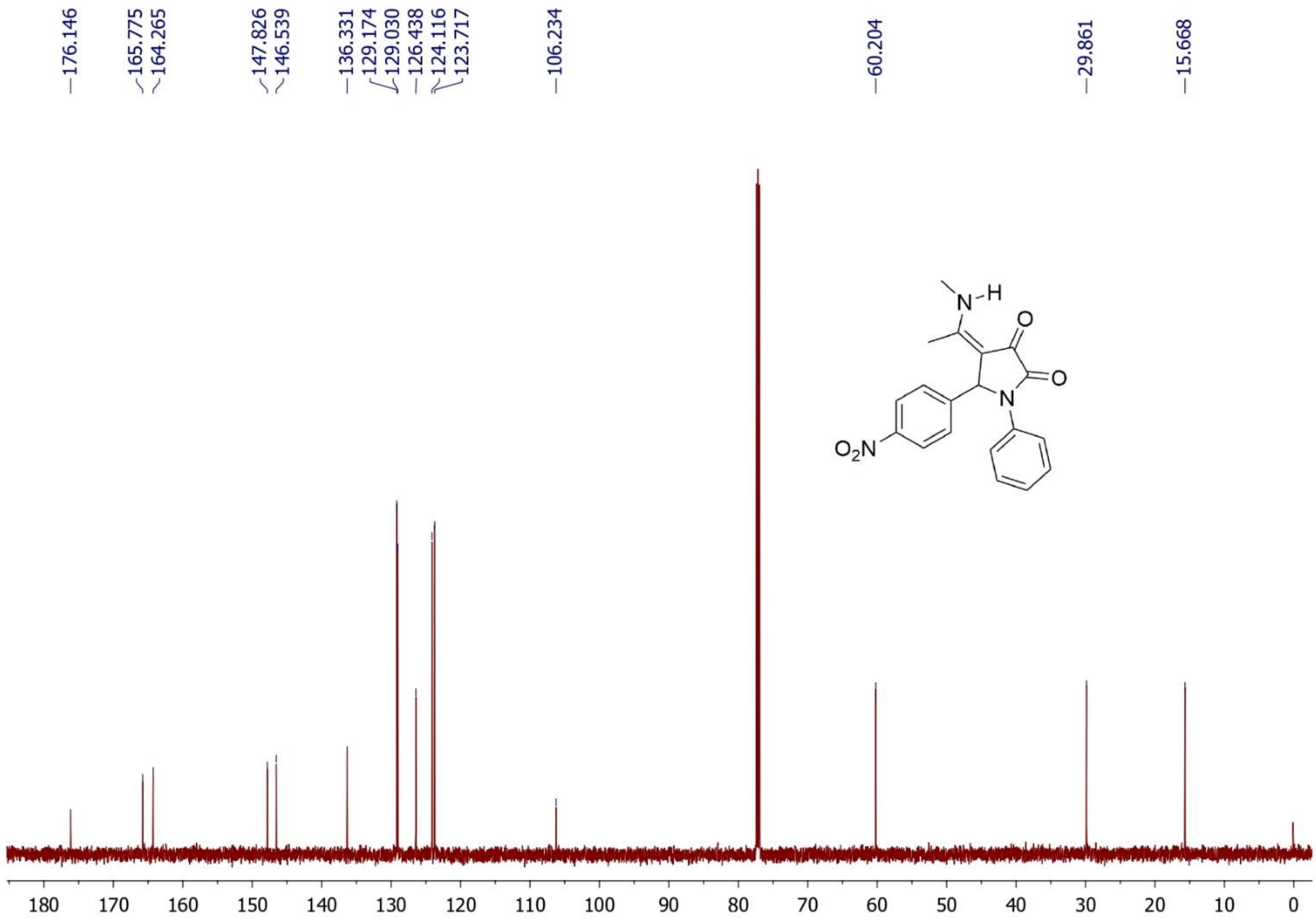

Figure S18. ^{13}C NMR spectrum of 4-(1-methylamino)ethylidene-1-phenyl-5-(4-nitrophenyl)pyrrolidine-2,3-dione (**5c**)

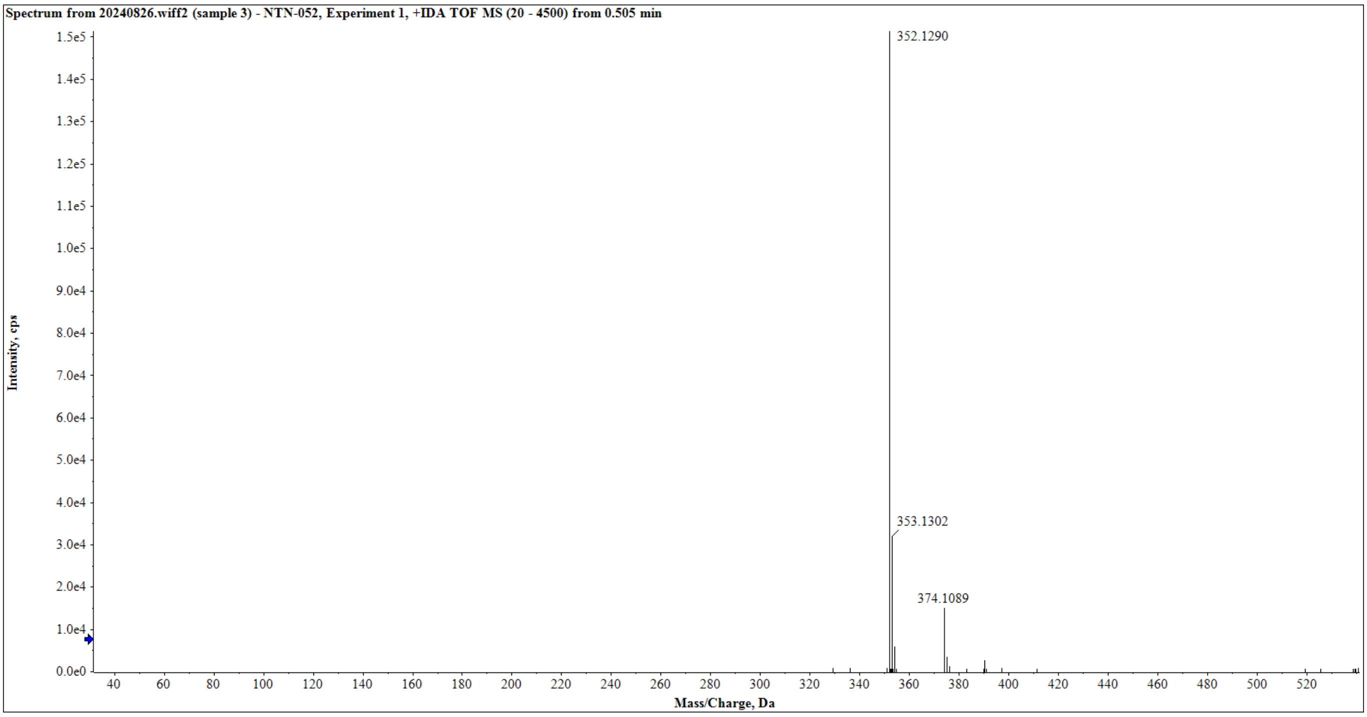

Figure S19. ESI-HRMS spectrum of 4-(1-methylamino)ethylidene-1-phenyl-5-(4-nitrophenyl)pyrrolidine-2,3-dione (**5c**)

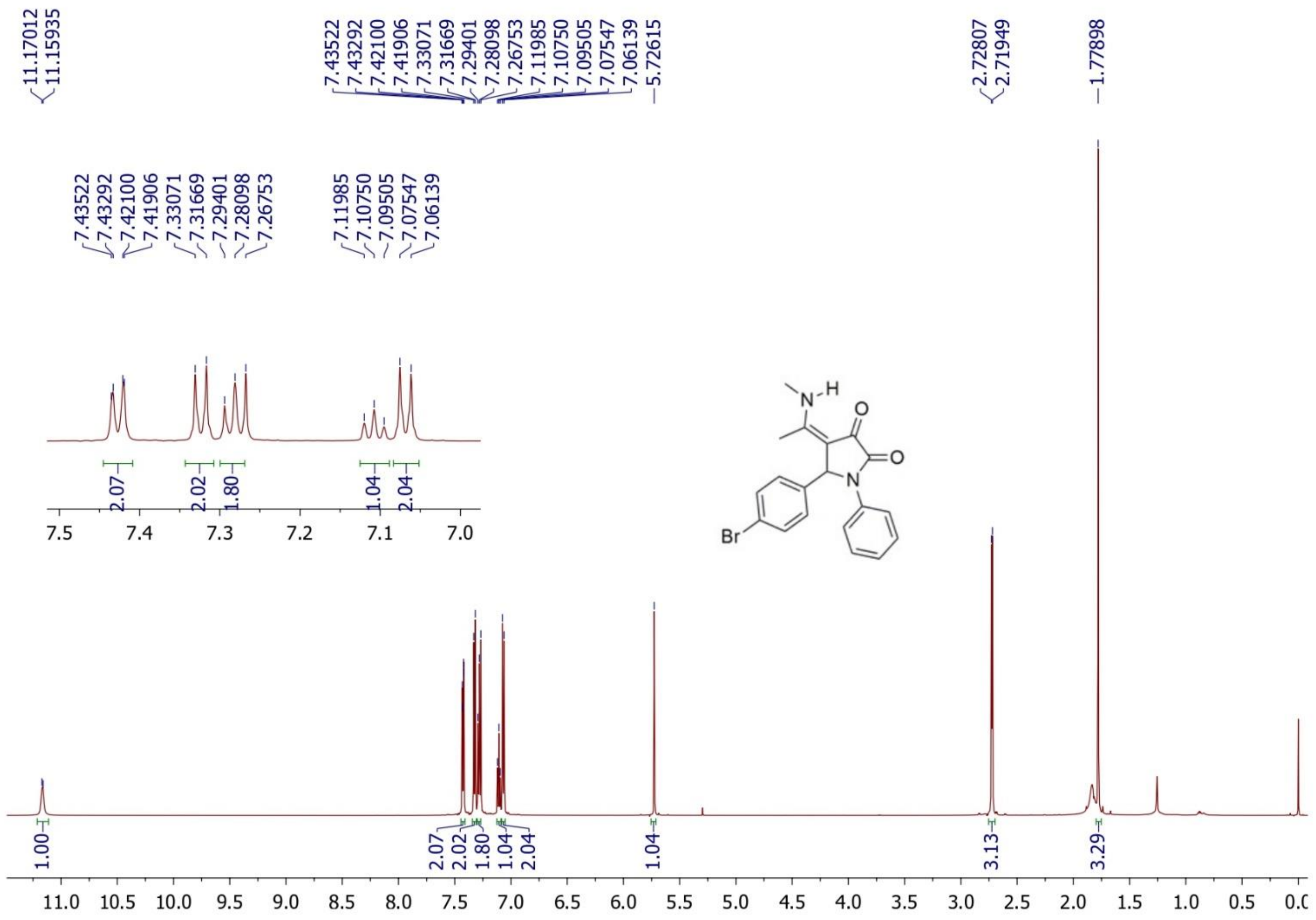

Figure S20. ^{1}H NMR spectrum of 4-(1-methylamino)ethylidene-1-phenyl-5-(4-bromophenyl)pyrrolidine-2,3-dione (**5d**)

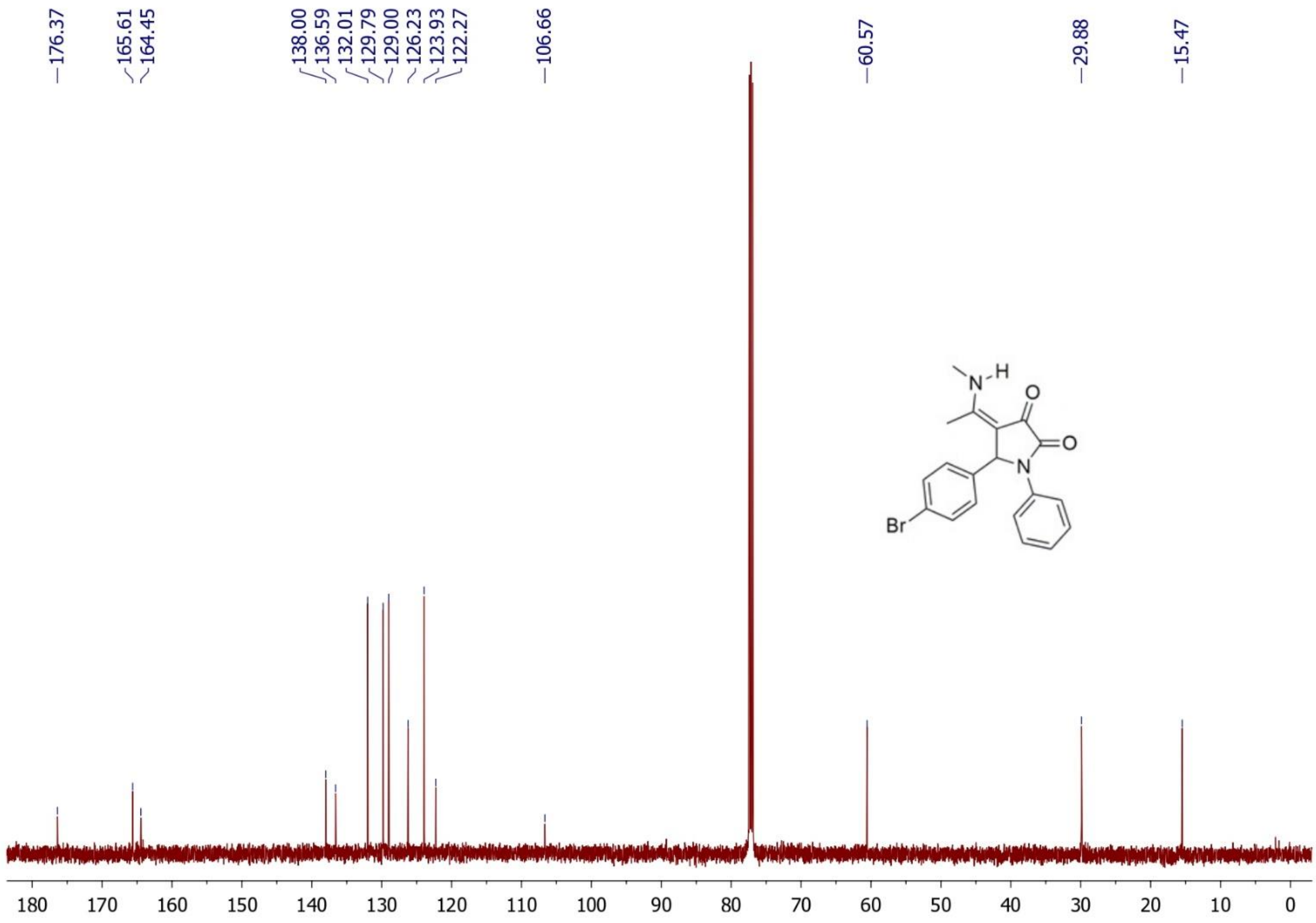

Figure S21. ^{13}C NMR spectrum of 4-(1-methylamino)ethylidene-1-phenyl-5-(4-bromophenyl)pyrrolidine-2,3-dione (**5d**)

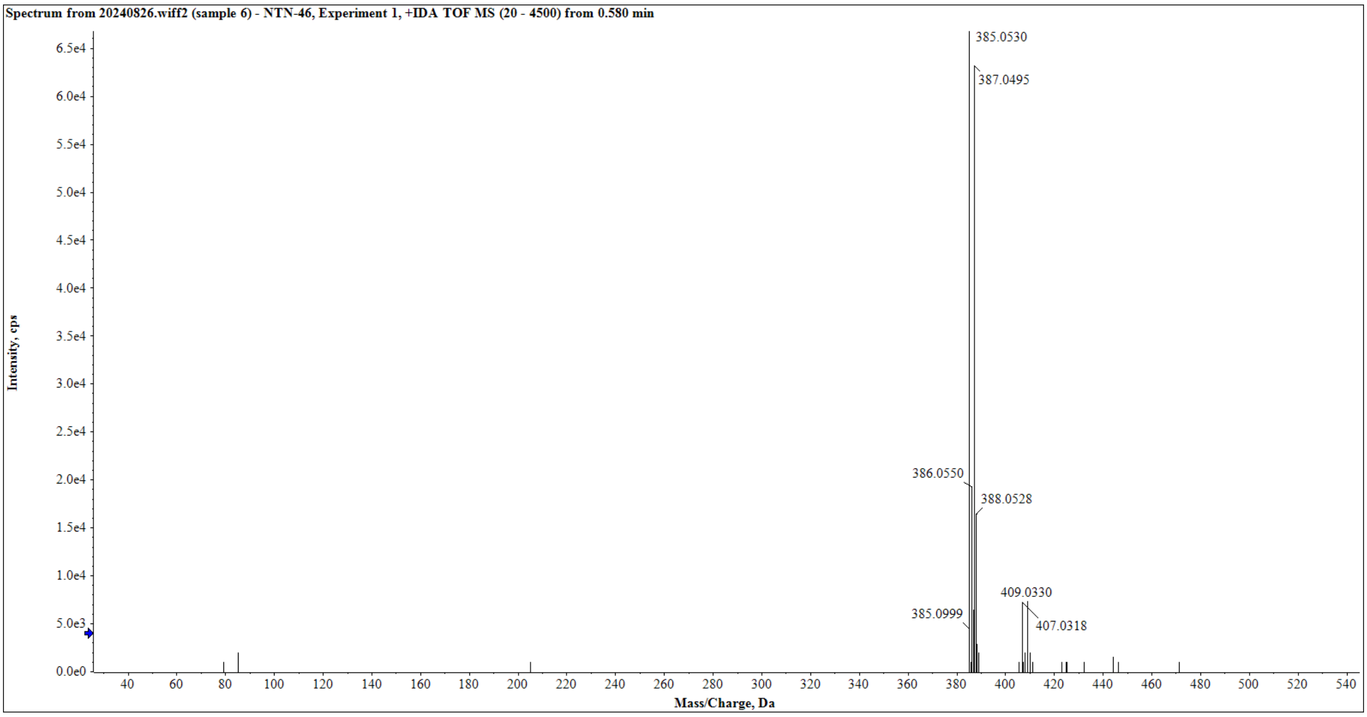

Figure S22. ESI-HRMS spectrum of 4-(1-methylamino)ethylidene-1-phenyl-5-(4-bromophenyl)pyrrolidine-2,3-dione (**5d**)

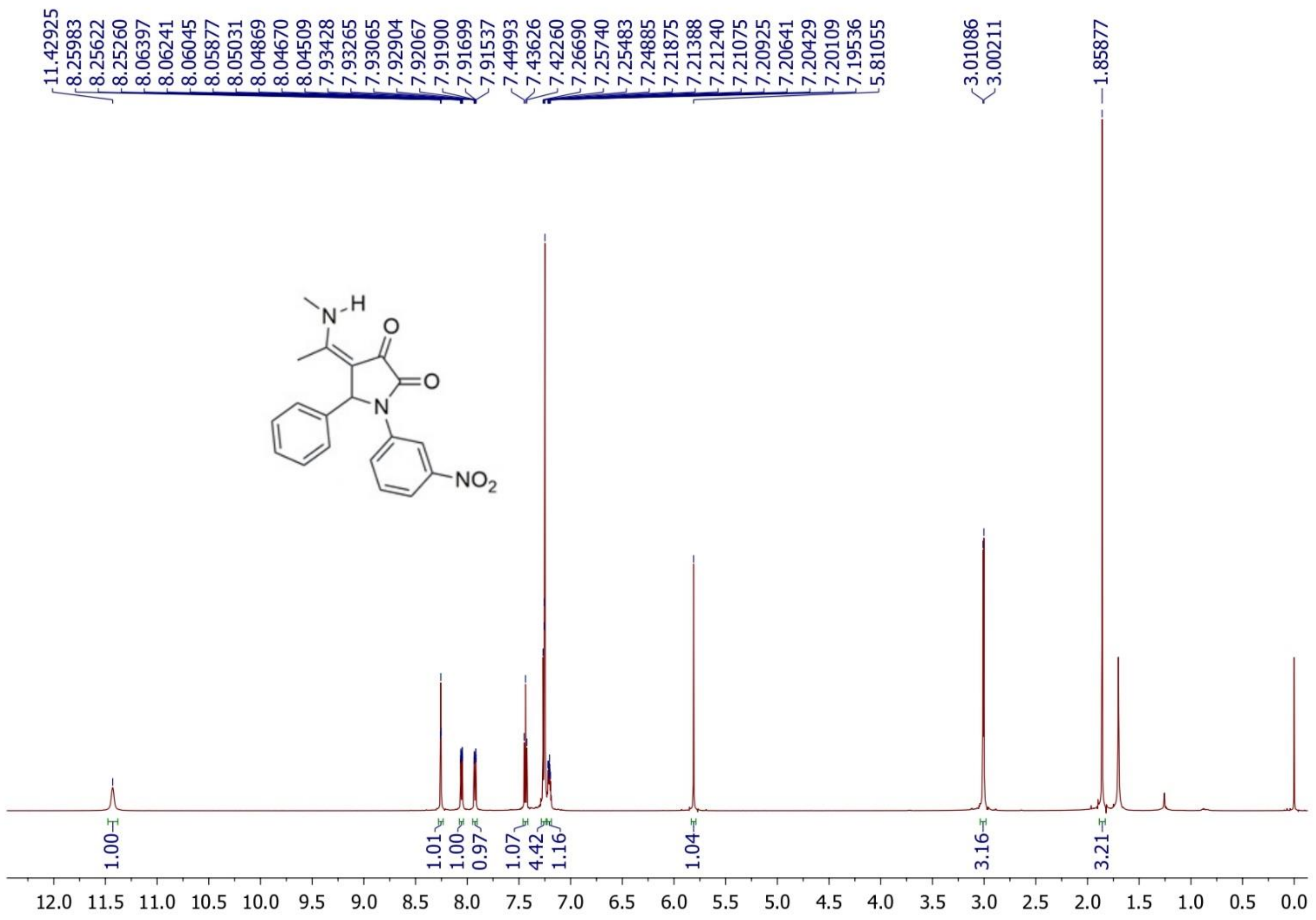

Figure S23. ^{1}H NMR spectrum of 4-(1-methylamino)ethylidene-5-phenyl-1-(3-nitrophenyl)pyrrolidine-2,3-dione (**5e**)

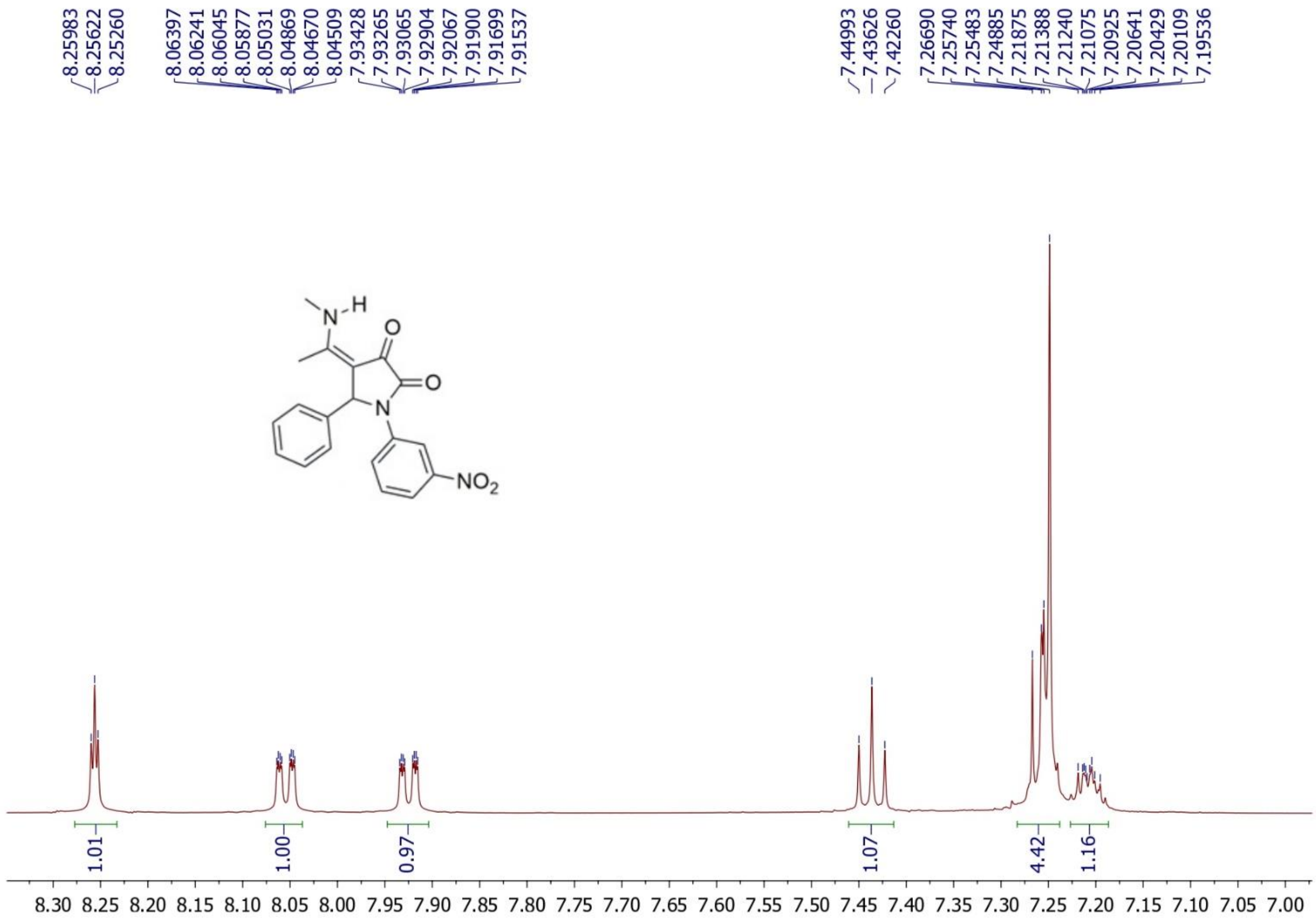

Figure S24. ^{1}H NMR spectrum of 4-(1-methylamino)ethylidene-5-phenyl-1-(3-nitrophenyl)pyrrolidine-2,3-dione (**5e**) in the chemical shift region of 7.00 ppm to 8.30 ppm

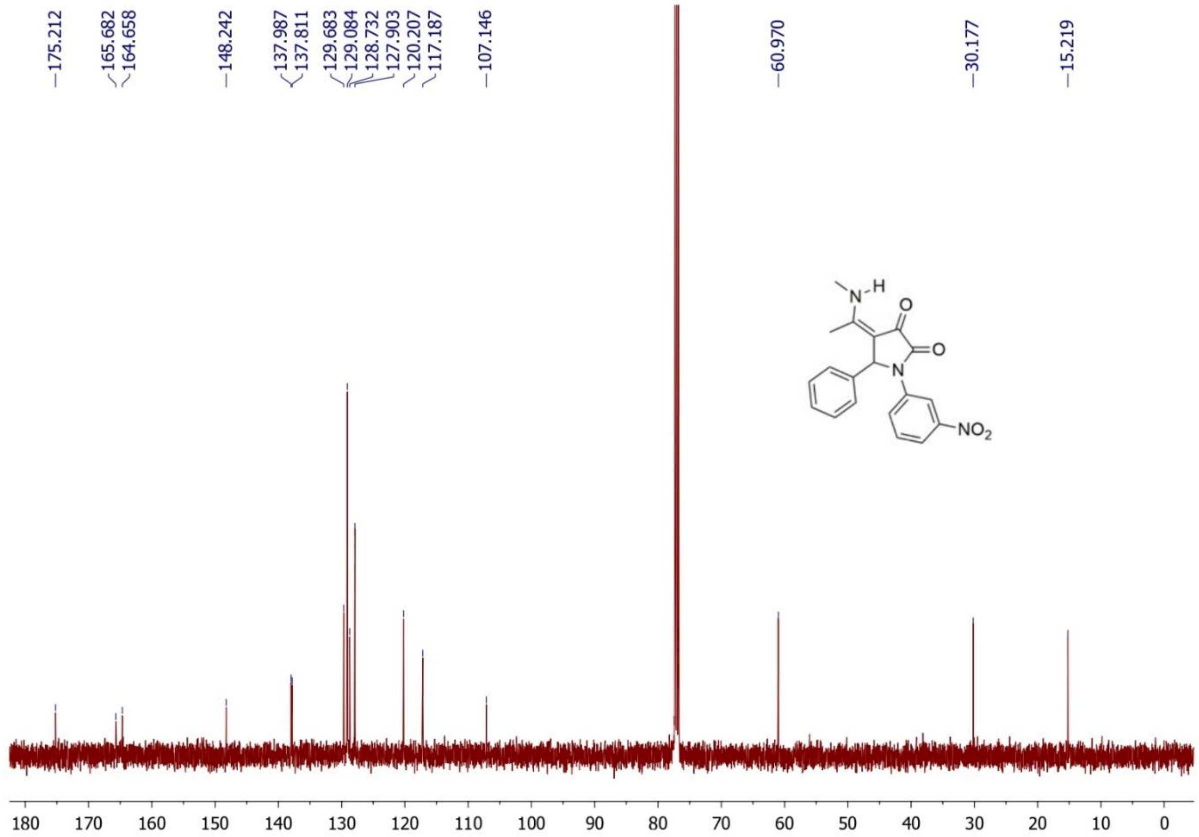

Figure S25. ^{13}C NMR spectrum of 4-(1-methylamino)ethylidene-5-phenyl-1-(3-nitrophenyl)pyrrolidine-2,3-dione (**5e**)

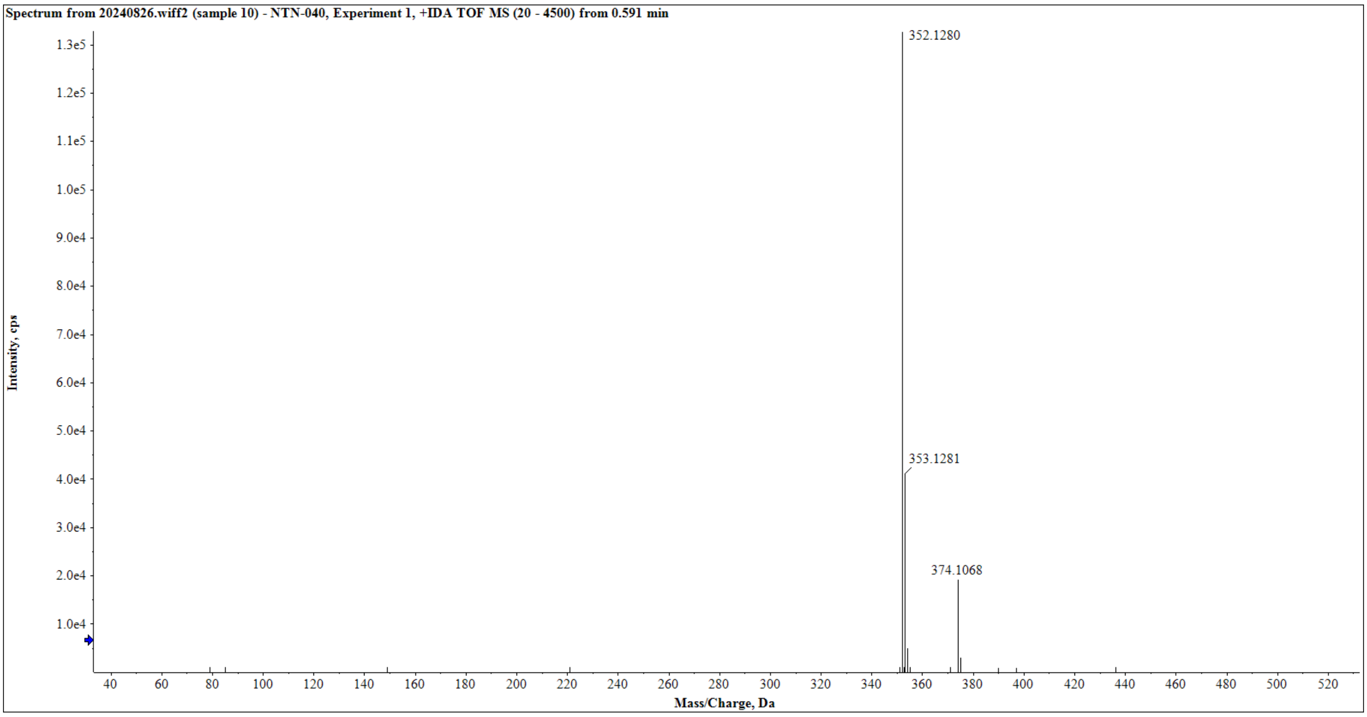

Figure S26. ESI-HRMS spectrum of 4-(1-methylamino)ethylidene-5-phenyl-1-(3-nitrophenyl)pyrrolidine-2,3-dione (**5e**)
